# The epidemiology of hepatitis C virus in Egypt: a systematic review and data synthesis

**DOI:** 10.1186/1471-2334-13-288

**Published:** 2013-06-24

**Authors:** Yousra A Mohamoud, Ghina R Mumtaz, Suzanne Riome, DeWolfe Miller, Laith J Abu-Raddad

**Affiliations:** 1 Infectious Disease Epidemiology Group, Weill Cornell Medical College - Qatar, Cornell University, Qatar Foundation - Education City, Doha, Qatar; 2 Department of Public Health, Weill Cornell Medical College, Cornell University, New York, New York, USA; 3 Vaccine and Infectious Disease Division, Fred Hutchinson Cancer Research Center, Seattle, Washington, USA; 4 Department of Tropical Medicine, Medical Microbiology and Pharmacology, John A. Burns School of Medicine, University of Hawaii, Honolulu, HI 96813, Hawaii

**Keywords:** Hepatitis C Virus, Epidemiology, Prevalence, Incidence, Egypt, Systematic Review

## Abstract

**Background:**

Egypt has the highest prevalence of hepatitis C virus (HCV) in the world, estimated nationally at 14.7%. Our study’s objective was to delineate the evidence on the epidemiology of HCV infection among the different population groups in Egypt, and to draw analytical inferences about the nature of HCV transmission in this country.

**Methods:**

We conducted a systematic review of all data on HCV prevalence and incidence in Egypt following PRISMA guidelines. The main sources of data included PubMed and Embase databases. We also used a multivariate regression model to infer the temporal trend of HCV prevalence among the general population and high risk population in Egypt.

**Results:**

We identified 150 relevant records, four of which were incidence studies. HCV incidence ranged from 0.8 to 6.8 per 1,000 person-years. Overall, HCV prevalence among pregnant women ranged between 5-15%, among blood donors between 5-25%, and among other general population groups between 0-40%. HCV prevalence among multi-transfused patients ranged between 10-55%, among dialysis patients between 50-90%, and among other high risk populations between 10% and 85%. HCV prevalence varied widely among other clinical populations and populations at intermediate risk. Risk factors appear to be parenteral anti-schistosomal therapy, injections, transfusions, and surgical procedures, among others. Results of our time trend analysis suggest that there is no evidence of a statistically significant decline in HCV prevalence over time in both the general population (p-value: 0.215) and high risk population (p-value: 0.426).

**Conclusions:**

Egypt is confronted with an HCV disease burden of historical proportions that distinguishes this nation from others. A massive HCV epidemic at the national level must have occurred with substantial transmission still ongoing today. HCV prevention in Egypt must become a national priority. Policymakers, and public health and medical care stakeholders need to introduce and implement further prevention measures targeting the routes of HCV transmission.

## Background

The Egyptian Demographic Health Survey (EDHS), a cross sectional survey including hepatitis C virus (HCV) biomarkers, was conducted in 2008 on a large nationally representative sample [1]. It estimated HCV prevalence among the 15–59 years age group to be 14.7% [1]. Accordingly, Egypt has the highest HCV prevalence in the world [2-4]. This unparalleled level of exposure to this infection appears to reflect a national level epidemic. It has been postulated that the epidemic has been caused by extensive iatrogenic transmission during the era of parenteral-antischistosomal-therapy (PAT) mass-treatment campaigns [5,6]. Today, HCV infection and its complications are among the leading public health challenges in Egypt [7].

Multiple community- and facility-based studies were conducted among different population groups in Egypt over the last two decades to assess the distribution of infection in the population. These studies have immensely improved our understanding of HCV epidemiology in Egypt. Nevertheless, two lingering and critical questions regarding HCV transmission in Egypt are yet to be addressed satisfactorily:

1) Does the high HCV prevalence reflect mainly historical exposures during the PAT campaigns before 1985, with limited current infection incidence?

2) If not, to what extent is HCV transmission still ongoing, and what are the drivers, risk factors, and modes of this transmission?

The objective of our study is to attempt, at least in part, to address these two questions through a comprehensive systematic review and integrated analysis of multiple sources of data about HCV prevalence and incidence in Egypt. Our study examined side-by-side information collected by different methods, by different investigators, and in different populations, allowing us to corroborate hypotheses across datasets, thereby reducing the impact of potential biases that can exist in a single study, dataset, or line of evidence. Our approach also facilitated an identification of the key research, policy, and programming priorities that require further investigation and consideration.

## Methods

### Data sources and search strategy

We conducted a systematic review of the prevalence and incidence of HCV in the different population groups in Egypt following the PRISMA guidelines [8]. The PRISMA checklist can be found in Table S1, see Additional file 1. The main data sources for this investigation were: PubMed (Medline) and Embase databases. The above data sources were searched with no time or language restrictions. PubMed and Embase were searched using both MeSH/*Emtree* terms, respectively, and text terms. MeSH/*Emtree* terms were exploded to cover all subheadings. Details of the search criteria for each of these databases can be found below:

PubMed: ((“Hepatitis C”[Mesh] OR “Hepatitis C Antibodies”[Mesh] OR “Hepatitis C Antigens”[Mesh] OR “Hepacivirus”[Mesh] OR “Hepatitis C, chronic/epidemiology”[Mesh] OR “Hepatitis C, chronic/etiology”[Mesh] OR “Hepatitis C, chronic/transmission”[Mesh] OR “Hepatitis C, chronic/virology”[Mesh] OR “Hepatitis C”[Text] OR “HCV”[Text]) AND (“Egypt”[Mesh] or Egypt[Text])).

Embase: (egypt.mp. or exp Egypt/) and (exp hepatitis C/ or exp Hepatitis C virus/ or hepatitis C.mp. or HCV.mp. or hepacivirus.mp.).

### Ethics statement

Our study did not need an ethics committee approval or written informed consent because it relies entirely on published data.

### Study selection

The results of the searches were imported to a reference manager, Endnote, where duplicate publications were identified and excluded. The remaining unique and potentially relevant records were then imported into Microsoft Excel where screening for relevance and eligibility took place. The titles and abstracts of all records retrieved were screened for relevance independently by two of the authors (YM and SR). Screening for relevance was conducted in two stages: 1) Stage 1 involved screening all titles and abstracts to exclude all non-relevant articles; 2) Stage 2 involved retrieving and screening the full-text of all articles deemed relevant after the initial abstract screening, to further exclude any remaining non-eligible articles. Inconsistencies between reviewers were discussed and sorted out by consensus.

A publication was considered eligible for inclusion in the review if it had data on at least one of the following outcomes of interest: 1) prevalence of HCV as detected by HCV antibodies; and 2) incidence of HCV as detected by HCV antibodies. Only studies reporting primary data were included. Reviews of literature were excluded, but all data reported were checked and compared to the results of our search. Any additional study identified in the excluded review and not retrieved by our search was identified and added to our review. Case reports and case series were excluded. All other study designs were eligible for inclusion. Distinction was made between the number of “reports” (actual publications i.e. papers, conference abstracts etc.) and the number of “studies” (actual study and research project). Multiple reports of the same study were identified as duplicates and counted as one study.

Eligible studies were then categorized into two types: prevalence studies and incidence studies. Any article reporting both the prevalence and incidence of HCV was counted as two studies, one for incidence and one for prevalence. Results were then pooled into one list containing all eligible and unique studies.

### Data extraction and population classification

The following data were then extracted from each eligible study included in the review: author, year of data collection, year of publication, city, study site, study design, sampling technique, population (blood donors, barbers, health care workers, injecting drug users (IDUs) etc.), socio-demographic characteristics of the population (sex, age, rural vs. urban etc.), sample size, and prevalence and/or incidence of HCV. Although our search criteria did not specifically target publications reporting HCV RNA prevalence or risk factors in Egypt, we extracted this information from eligible publications when available. Risk factors were extracted only if they were statistically significant in a multivariate regression analysis within a study that was found relevant according to our search criteria.

Data on the above mentioned indicators were extracted from included records and entered into a computerized database on Microsoft Excel. Extracted data were then classified and analyzed on the basis of the study population’s risk of acquiring HCV. The four defined major population risk groups are:

1) Populations at direct or high risk: this group includes IDUs, multi-transfused patients such as hemophiliacs and thalassemics, dialysis patients, and viral hepatitis patients, among others.

2) Populations at indirect or intermediate risk: this group includes *familial contacts* of HCV patients i.e. their children, spouses, and other household contacts; select *practitioners of professions at risk of HCV* such as dentists, healthcare workers and barbers; *populations with potential IDU exposures* including: prisoners and HIV patients; *and populations with health facility/injecting exposures* such as diabetic patients and hospitalized populations.

3) General population groups (populations which are not at an elevated risk of HCV exposure): pregnant women, blood donors, children, rural populations, army recruits or fire brigade personnel, outpatient clinic attendees, populations defined in case–control studies as healthy populations (controls), among other groups categorized together as “other general population” groups.

4) Special clinical population groups such as Hodgkin’s lymphoma (HL) patients, lichen planus (LP), and liver disease patients, among others. This category includes patients with specific diseases that require clinical care, and thus can be exposed to HCV in medical care facilities, though at variable levels of risk that is difficult to categorize among any of the above mentioned population groups.

Within any specific category of the above population groups, considerable heterogeneity and different subgroup trends may exist.

In order to create prevalence figures, HCV prevalence measures within each of the population groups above were stratified into two strata: pre-2001 and post-2001, based on the year in which the study was conducted. The year 2001 was chosen as the cut-off year, as this was the year in which the Egyptian Ministry of Health initiated broad infection control programs in the country [9].

### Time trend analysis

We conducted a time trend analysis investigating the change in HCV prevalence among the general population and high risk population in Egypt with respect to time. We started by conducting univariate linear regression analyses examining the change in HCV prevalence over time in each of the general population subgroups separately. A similar analysis was conducted in each of the high risk population subgroups. To avoid systematic biases in any one subgroup and to increase the statistical power of the time trend analysis, we also used a multivariate linear regression model estimating the temporal trend in HCV prevalence, while adjusting for the different subgroups in each population grouping. Two such models were performed: one for the general population and the other for the high-risk population. Mean HCV prevalence was modeled using a model that includes as predictors: time (in years) and the different general population subgroups (or high risk population subgroups).

The general population regression model stipulates that:

$$\begin{array}{ll} \mathrm{Mean} & \mathrm{HCV}\;\mathrm{Prevalence}=\beta_{0}+\beta_{1}*\mathrm{Time}\\ & +\beta_{2}*\mathrm{Antenatal}\;\mathrm{clinic}\;\mathrm{attendees}\\ & +\beta_{3}*\mathrm{Blood}\;\mathrm{donors}\;+\beta_{4}*\mathrm{Rural}\;\mathrm{village}\;\mathrm{residents}\\ & +\beta_{5}*\mathrm{Children}+\beta_{6}*\mathrm{Healthy}\;\mathrm{populations}\\ & +\beta_{7}*\mathrm{Army}\;\mathrm{recruits}+\beta_{8}*\mathrm{Other} \end{array}$$

Similarly, the high risk population regression model stipulates that:

$$\begin{array}{ll} \mathrm{Mean}\; & \mathrm{HCV}\;\mathrm{Prevalence}=\beta_{0}+\beta_{1}*\mathrm{Time}\\ & +\beta_{2}*\mathrm{Multi-transfused}\;\mathrm{patients}\\ & +\beta_{3}*\mathrm{Schistosomiasis}\;\mathrm{patients}\\ & +\beta_{4}*\mathrm{Thalassemic}\;\mathrm{patients}\\ & +\beta_{5}*\mathrm{Viral}\;\mathrm{hepatitis}\;\mathrm{patients} \end{array}$$

In both models, *βi* are the parameters of the statistical model and Time and sub-population names are the indicator variables.

Data used for these analyses were extracted from the eligible studies included in this review. In the presence of both an overall HCV prevalence measure as well as stratified prevalence measures, we included only the overall prevalence measure. Including both overall and stratified prevalence measures from the same study would have given more weight to those studies, relative to others, thereby biasing our results. The year of data collection was estimated for studies missing this variable. This was done by conducting a correlation analysis between the year of data collection and the year of publication for studies having data on both, followed by a paired t-test to estimate the mean difference between them. We then applied this difference to the year of publication to estimate the year of data collection when missing. Midpoints were calculated and used for studies conducted over a number of years. The statistical analysis was conducted using STATA version 11 (STATA corporation, College Station, Texas).

## Results

### Search results

**Figure 1:**
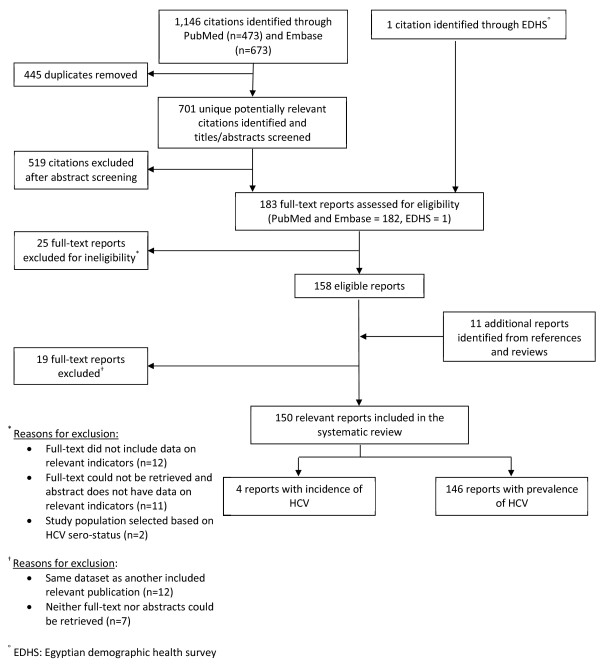
**Flow of article selection for the HCV prevalence and incidence in Egypt search.** This chart, adapted from the PRISMA 2009 flow diagram, displays the flow of article selection for the HCV incidence and prevalence in Egypt search of scientific databases, namely PubMed and Embase.

The study selection process is described in Figure 1, as adapted from the PRISMA 2009 flow diagram [8]. The number of records retrieved through both PubMed and Embase was 1,146 as of August 1, 2012, out of which 445 were excluded as duplicates. After assessing all documents according to their titles and abstracts, the full-text of 183 records were retrieved for screening in addition to 11 papers identified through references of reviews. Of those, 150 records were found eligible for inclusion in the present article. Only one relevant record was identified outside the PubMed and Embase search; the EDHS [1]. No single report has reported both a relevant incidence measure *and* a relevant prevalence measure.

### HCV incidence

Five incidence measures were identified through our search, reported in four incidence reports (Table 1). All studies were conducted in rural areas of Egypt among village residents, pregnant women, and children [10-13]. Overall high HCV incidence rates were observed in these studies ranging from 0.8 to 6.8 per 1,000 person-years.

**Table 1:** Studies reporting hepatitis C virus incidence in Egypt

**Citation**	**Year**	**Location**	**Study population**	**Sample size**	**Incidence (per 1,000 person-years)**
Mohamed,05 [14]	1997-2000	Qalubyia, Lower Egypt	Village residents	2,463	6.8
Mohamed,05 [14]	1997-2000	Assuit, Upper Egypt	Village residents	4,275	0.8
Saleh,08 [15]	1997-2006	Menoufia, Lower Egypt	Pregnant women	2,177	5.2
Mostafa,10 [16]	2001-2003	Menoufia, Lower Egypt	Village residents	3,580	2.4
Saleh,10 [17]	2000-2006	Menoufia, Lower Egypt	Children of 3 villages with high prevalence of HCV	2,852	2.7

### Prevalence of HCV in the general population

Sixty-nine studies reported HCV prevalence in the general population. Details are shown in Table 2. HCV prevalence among the general population of Egypt is documented to be very high. The 2008 EDHS measured HCV prevalence to be 14.7% among a nationally representative sample of 11,126 Egyptians aged 15–59 years old [1]. The diverse HCV studies conducted among different general population subgroups, regardless of design or methodology, consistently report a very high HCV prevalence, as high as 41% in some studies [10]. Overall, the prevalence appears to increase dramatically with age with the highest rates observed among populations aged greater than 40 years.

**Table 2:** Studies reporting hepatitis C virus prevalence among the general population in Egypt

**Citation**	**Year**	**Location**	**Sampling**	**Population characteristic**	**Sample size**	**Sero-prevalence**	**RNA prevalence**
**Outpatient clinic attendees**							
Khalifa,93 [18]	1990-1	Cairo city, Cairo	CS	Children	84	0.0%	N/A
El-Nanawy,95 [19]	N/A	Alexandria city, Alexandria	CS	Children	110	11.8%	N/A
Miras,02 [20]	N/A	Tanta, Gharbia, Lower Egypt	CS	In/out-patient children living in HCV endemic region	105	0.0%	N/A
El-Raziky,07 [21]	2004	Cairo city, Cairo	CS	Children 1–9 yrs old	1,042	1.4%	0.5%
Kandil,07 [22]	2004-6	Cairo city, Cairo	CS	Healthy children	20	5.0%	N/A
**Antenatal clinic attendees**							
Hassan,93 [23]	N/A	N/A	CS		1,536	4.3%	N/A
Agha,98 [24]	1996-7	Mansoura, Dakahlia, Lower Egypt	CS		767	13.7%	23.7%
Kassem,00 [25]	1996	Alexandria city, Alexandria	CS		100	19.0%	14.0%
Stoszek,05 [26]	1997-03	3 rural villages in Nile River Delta, Lower Egypt	CS		2,587	15.8%	10.8%
Zahran,10 [27]	2008-9	Assuit, Upper Egypt	CS		500	8.0%	7.4%
Abdulqawi,10 [28]	2003-8	Benha, Qalubiya, Lower Egypt	CS		1,224	8.6%	6.8%
Abo Elmagd,11 [29]	N/A	N/A	CS	20-40 year old mothers	61	13.0%	N/A
**Blood donors**							
Kamel,92 [30]	1992	Cairo city, Cairo	CS	Male university students, 20–27 years	2,164	9.7%	N/A
El-Zayadi,92 [31]	N/A	N/A	CS		76	5.2%	N/A
Darwish,92 [32]	N/A	N/A	CS		90	14.4%	N/A
Darwish,93 [33]	1992	Cairo city, Cairo	CS		163	13.6%	N/A
El-Ahmady,94 [10]	N/A	N/A	CS	Paid blood donors	99	35.4%	N/A
Quinti,95 [34]	1992-4	Alexandria city, Alexandria	CS		283	20.8%	N/A
Bassily,95 [35]	N/A	Cairo city, Cairo	CS		188	26.6%	N/A
El Gohary,95 [36]	1990-2	Suez city, Suez and Ismailia, Lower Egypt	CS		1,187	14.5%	N/A
Attia,96 [37]	N/A	Cairo city, Cairo	CS		156	21.8%	N/A
Arthur,97 [38]	1993	24 Governorates	CS		2,644	24.8%	N/A
El-Zayadi,97 [39]	N/A	N/A	CS		180	9.4%	N/A
Gad,01 [40]	1998	Ismailia, Lower Egypt	CS		20	20.0%	N/A
Tanaka,04 [41]	1999	13 governorates, Upper and Lower Egypt	CS		3,608	8.8%	6.2%
Hashish,05 [42]	N/A	Alexandria city, Alexandria	CS		95	23.2%	N/A
El-Gilany,06 [43]	2002-3	Mansoura, Dakahlia, Lower Egypt	CS	Student voluntary blood donors	2,157	2.7%	N/A
Agha,06 [44]	N/A	N/A	CS		2,400	8.0%	N/A
El Damaty,07 [45]	2001	Cairo city, Cairo	CS		2,845	7.6%	N/A
El-Zayadi,08 [46]	2005	26 governorates	Random	All blood donors	760	5.0%	N/A
El-Zayadi,08 [46]	2005	26 governorates		Female blood donors	124	6.5%	N/A
El-Zayadi,08 [46]	2005	26 governorates		Male blood donors	636	4.7%	N/A
Ismail,09 [47]	2000-7	Mansoura, Dakahlia, Lower Egypt	CS		55,922	12.0%	N/A
Ashour,09 [48]	2006-8	8 governorates	CS		515,758	4.8%	N/A
Elkareh,09 [11]	2008	Menoufia, Lower Egypt	CS	Family replacement blood donors	4,709	12.7%	N/A
Elkareh,09 [11]	2008	Menoufia, Lower Egypt	CS	Blood donors	3,569	6.3%	N/A
Elkareh,09 [11]	2008	Menoufia, Lower Egypt	CS	Family replacement blood donors	8,705	14.6%	N/A
Elkareh,09 [11]	2008	Menoufia, Lower Egypt	CS	Blood donors	414	8.7%	N/A
Rushdy,09 [13]	2006-7	Suez Canal area	CS	All blood donors	9,150	5.6%	N/A
Rushdy,09 [13]	2006-7	Suez Canal area		Male blood donors	7,155	2.9%	N/A
Rushdy,09 [13]	2006-7	Suez Canal area		Female blood donors	1,995	1.7%	N/A
Eita,09 [12]	2005-8	Dakhilia, Lower Egypt	CS	Voluntary blood donors	73,431	4.6%	N/A
Eita,09 [12]	2005-8	Dakhilia, Lower Egypt	CS	Family blood donors	113,504	5.5%	N/A
Khattab,10 [36]	2000-8	Minya, Lower Egypt	CS		211,772	9.0%	N/A
Radwan,10 [49]	2009	N/A	CS		27,537	4.0%	N/A
Wasfi,11 [50]	2007-8	Alexandria city, Alexandria	CS		3,420	3.5%	N/A
Awadalla,11 [51]	N/A	Cairo city, Cairo	CS		1,000	16.8%	N/A
Farawela,12 [52]	2010-1	Cairo city, Cairo	CS		100	5%	N/A
**Rural village residents**							
Abdel-Wahab,94 [53]	1992	Menoufia, Lower Egypt	CS		270	18.1%	N/A
Kamel,94 [54]	1992	Sada, Kafr El Sheikh, Lower Egypt	All village residents		1,259	15.9%	N/A
El Gohary,95 [36]	1990-2	Suez Canal area	CS	Healthy blood volunteers resident to rural area with high schistosomiasis	271	14.4%	N/A
El Gohary,95 [36]	1990-2	North Sinai, frontier	CS	Bedouin population with low schistosomiasis	148	15.5%	N/A
Darwish,95 [55]	N/A	N/A	CS	Healthy villagers and non-professional blood donors	188	21.8%	N/A
Darwish,96 [56]	1994	Kalama, Qaluobyia, Lower Egypt	CS	Village residents:1–3 years	12	0.0%	N/A
Darwish,96 [56]	1994	Kalama, Qaluobyia, Lower Egypt	CS	Village residents: 4–9 years	21	0.0%	N/A
Darwish,96 [56]	1994	Kalama, Qaluobyia, Lower Egypt	CS	Village residents: 10–19 years	46	8.0%	N/A
Darwish,96 [56]	1994	Kalama, Qaluobyia, Lower Egypt	CS	Village residents: 20–39 years	29	20.0%	N/A
Darwish,96 [56]	1994	Kalama, Qaluobyia, Lower Egypt	CS	Village residents: 40–67 years old	47	51.0%	N/A
El-Sayed,97 [57]	1993-4	Sinai, frontier	CS	Immigrants to a newly reclaimed area endemic for schistosomiasis	506	10.3%	N/A
Nafeh,00 [58]	N/A	Assuit, Upper Egypt	CS	Village residents ≥ 5 years of age (overall)	6,031	8.7%	5.4%
Nafeh,00 [58]	N/A	Assuit, Upper Egypt	CS	Village residents ≤30 years	4,164	3.6%	2.0%
Nafeh,00 [58]	N/A	Assuit, Upper Egypt	CS	Village residents >30 years	1,867	20.0%	12.9%
Abdel-Aziz,00 [59]	1997	Aghour El Soughra, Qaluobyia, Lower Egypt	CS	Village residents ≥5 years old (overall)	3,999	24.3%	14.8%
Abdel-Aziz,00 [59]	1997	Aghour El Soughra, Qaluobyia, Lower Egypt	CS	Village residents: ≤20 years	2,105	9.3%	N/A
Abdel-Aziz,00 [59]	1997	Aghour El Soughra, Qaluobyia, Lower Egypt	CS	Village residents >20 years	1,894	41.0%	N/A
Darwish,01 [60]	1994-5	Kalama, Qaluobyia, Lower Egypt	CS	Village residents >10 years of age	796	40.0%	N/A
El-Sadawy,04 [61]	N/A	Sharkia, Lower Egypt	CS		842	27.4%	7.4%
Arafa,05 [62]	2002-3	Zawiat Razin, Menoufia, Lower Egypt	CS		4,020	11.8%	N/A
Arafa,05 [62]	2002-3	Zawiat Razin, Menoufia, Lower Egypt	CS	Village residents: under 20 years old	1,759	2.8%	N/A
Arafa,05 [62]	2002-3	Zawiat Razin, Menoufia, Lower Egypt	CS	Village residents: 20 and over years old	2,252	18.9%	N/A
Mohamed,06 [63]	2002	Zawiat Razin, Menoufia, Lower Egypt	CS	Village residents 18–65 years of age	2,425	18.5%	N/A
Eassa,07 [64]	2006-7	Zagazig district, Sharkia, Lower Egypt	CS	Village households	304	10.9%	N/A
Aguilar,08 [65]	N/A	Fakkous and 8 surrounding villages	CS	Village residents: males	78	51.3%	38.5%
Aguilar,08 [65]	N/A	Fakkous and 8 surrounding villages, Sharkia, Lower Egypt	CS	Village residents: females	81	42.0%	29.6%
**Children**							
Abdel-Wahab,94 [53]	1992	Menoufia, Lower Egypt	CS	Rural male primary school children	190	12.1%	N/A
El-Sherbini,03 [66]	1994	N/A	CS	Village school children 6–15 years old	294	5.8%	2.4%
Mohamed,06 [67]	1997	Nile River Delta, Lower Egypt	CS	Village children 5–18 years old	1,823	8.2%	N/A
Mohamed,06 [67]	1997	Assuit, Upper Egypt	CS	Village children 5–18 years old	2,808	2.5%	N/A
El Sherbini,07 [68]	2002	Tanta, Gharbia, Lower Egypt	CS	School children	470	2.1%	0.8%
Barakat,11 [69]	2005	Alexandria city, Alexandria	PBS	School children	500	5.8%	4.4%
**Healthy individuals**							
El-Sayed,96 [70]	*1994*	South Sinai, frontier	CS	Tourism workers	740	14.3%	N/A
Mohamed,96 [71]	N/A	N/A	CS	Egyptians applying for work abroad	5,071	31.5%	N/A
Mohamed,96 [71]	N/A	N/A	CS	Egyptians applying for work abroad: Females	N/A	13.2%	N/A
Mohamed,96 [71]	N/A	N/A	CS	Egyptians applying for work abroad: Males	N/A	34.0%	N/A
Gohar,95 [72]	N/A	N/A	CS		15	13.3%	N/A
Halim,99 [73]	1996	Cairo city, Cairo	CS	Healthy staff of the university	50	6.0%	N/A
Hassan,01 [74]	1995-6	Cairo city, Cairo	CS	Healthy individuals visiting hospitalized friends	35	42.9%	N/A
Strickland,02 [75]	N/A	Nile River Delta, Lower Egypt	CS		212	46.7%	36.3%
El-sayed,06 [76]	2002	Cairo city, Cairo	CS		36	8.3%	2.8%
El Bassuoni,08 [77]	N/A	N/A	CS		10	30.0%	N/A
Salama,09 [78]	N/A	N/A	CS		20	5.0%	N/A
**Army recruits/Fire brigade personnel**							
Farghaly,93 [79]	N/A	N/A	CS	Army recruits	726	33.0%	N/A
Abdel-Wahab,94 [53]	1992	Lower Egypt	CS	Army recruits	300	22.1%	N/A
Quinti,95 [34]	1992-4	Alexandria city, Alexandria	CS	Fire brigade personnel	541	39.0%	N/A
**Other general populations**							
El-Ahmady,94 [10]	N/A	Cairo city, Cairo	CS		292	24.3%	N/A
El-Sadawy,04 [61]	N/A	Sharkia, Lower Egypt	PBS		1,422	25.8%	7.7%
El-Sadawy,04 [61]	N/A	Sharkia, Lower Egypt	PBS	General population in urban areas	580	23.4%	N/A
El-Sadawy,04 [61]	N/A	Sharkia, Lower Egypt	PBS	General population: <20 years	414	4.8%	N/A
El-Sadawy,04 [61]	N/A	Sharkia, Lower Egypt	PBS	General population: 20 – 30 years	163	14.1%	N/A
El-Sadawy,04 [61]	N/A	Sharkia, Lower Egypt	PBS	General population: 30 – 40 years	253	30.0%	N/A
El-Sadawy,04 [61]	N/A	Sharkia, Lower Egypt	PBS	General population: >40 years	592	41.9%	N/A
Mohamed,04 [80]	1996-7	10 governorates	PBS	General population	7,357	13.5%	N/A
El Zanaty,09 [1]	2008	Nationwide	PBS		11,126	14.7%	9.8%

*CS* convenience sampling, *PBS* probability-based sampling, *N/A* not available.

A number of studies were conducted among blood donors. A higher prevalence is observed among paid blood donors and family replacement blood donors compared to voluntary donors [10-12]. Male blood donors had a higher prevalence than their female counterparts [13]. Blood donors from rural areas had a higher prevalence than those from urban areas [38].

Multiple studies were conducted among village residents in high HCV prevalence areas (Table 2). The overall prevalence in rural areas averaged about 20%, higher than the national average. A study conducted in Kalama, a village in the Nile Delta, reported HCV prevalence of 40% among village residents [60]. Similar to blood donor studies, village residents were shown to have a higher prevalence among males compared to females [62,81], and a marked growth in prevalence with age [56,58,59]. A study conducted in 1997, among 3,993 residents of a village in the Nile Delta region, observed prevalence rates in children, ages 0–19, ranging between 7 and 9.9%. This rate increased to 27.6% in those 20–39 years and more than doubled to 56.7% among village residents greater than 40 years of age [82].

High HCV prevalence was also observed among pregnant women and children in Egypt. Recent studies conducted among pregnant women reported a prevalence of about 8% in Assuit [27] and Benha [28], and as high as 15.8% in rural villages of the Nile Delta [26]. Studies conducted among rural school children reported an average prevalence of about 7% [53,66,68,69], while the average prevalence in children attending outpatient clinics was found to be approximately 4% [18,19,21,22]. High prevalence was also observed among select subgroups such as tourism workers [70], army recruits [53,79] and fire brigade personnel [34].

**Figure 2:**
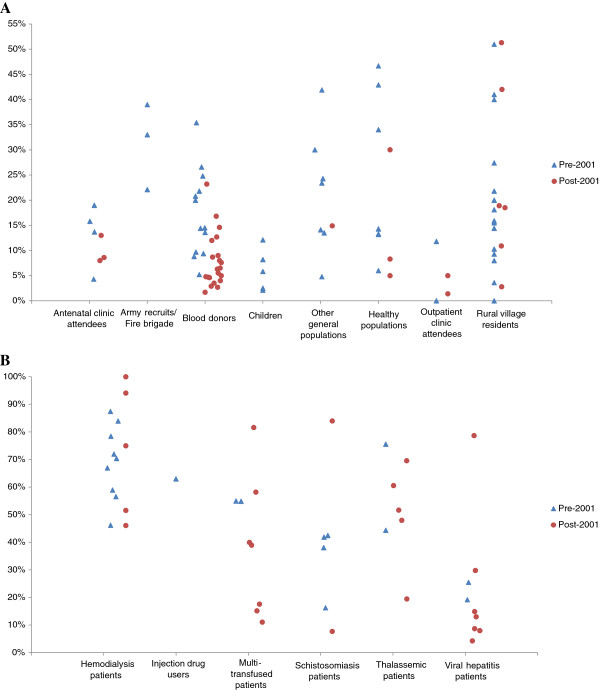
**Hepatitis C virus (HCV) prevalence among the general population and populations at direct or high risk in Egypt, in studies conducted pre- and post-2001. A**: Graph depicting HCV prevalence among different general population groups. **B**: Graph depicting HCV prevalence among different high/direct risk populations. In this figure, we included only stratified HCV prevalence measures, if these stratified measures were available. Otherwise, we included the overall prevalence measures in the study.

Figure 2A depicts the range of prevalence within each subgroup in studies conducted pre- and post-2001. Among blood donors, studies appear to cluster at lower HCV prevalence levels post- 2001 infection control programs, compared to pre-2001. However, no distinct pattern can be observed within each of the other subgroups.

### Prevalence of HCV among populations at direct or high risk of exposure

We classified populations at direct or high risk of HCV exposure into six subcategories: viral hepatitis patients, multi-transfused patients, thalassemia patients, schistosmiasis patients, patients on hemodialysis and IDUs. Table 3 lists all the studies in each sub-category and the reported prevalence measures.

**Table 3:** Studies reporting prevalence of hepatitis C virus among populations at direct or high risk of exposure in Egypt

**Citation**	**Year**	**Location**	**Sampling**	**Population characteristic**	**Sample size**	**Sero-prevalence**	**RNA prevalence**
**Viral hepatitis patients**							
El-Ahmady,94 [10]	N/A	Cairo city, Cairo	CS		51	25.5%	N/A
El-Gohary,94 [83]	N/A	Suez city, Suez	CS		140	19.2%	N/A
El Gaafary,05 [84]	2002	Cairo city, Cairo	CS		309	14.9%	N/A
Meky,06 [85]	2002-5	Nile River Delta, Lower Egypt	CS		47	78.7%	70.2%
Zakaria,07 [86]	2001-2	Giza, Upper Egypt	CS		200	13.0%	N/A
Kalil,10 [87]	2004-5	Assuit, Upper Egypt	CS	Children with viral hepatitis	150	8.7%	8.7%
Talaat,10 [88]	2001-4	Alexandria city, Alexandria/Abassia, Cairo/Mahalla, Gharbia, Lower Egypt/Qena and Aswan, Upper Egypt	CS		4,189	29.8%	N/A
Eldin,10 [89]	N/A	N/A	CS		235	4.3%	N/A
Badawy,12 [90]	N/A	Cairo city, Cairo	CS	Male military recruits with viral hepatitis	99	8%	N/A
**Multi-transfused patients**							
Khalifa,93 [18]	1990-1	Cairo city, Cairo	CS	Multi-transfused children	84	55.0%	N/A
Abdel-Wahab,94 [53]	1992	Cairo city, Cairo	CS	Multi-transfused children	71	54.9%	N/A
Said,09 [91]	N/A	Cairo city, Cairo	CS	Multi-transfused children with hematological disorders	49	81.6%	49.0%
Said,09 [91]	N/A	Cairo city, Cairo	CS	Multi-transfused children with hematological malignancies	51	17.6%	23.5%
Salama,09 [78]	N/A	Cairo city, Cairo	CS	Multi-transfused children	33	15.2%	N/A
Kalil,10 [87]	2004-5	Assuit, Upper Egypt	CS	Multi-transfused children	165	58.2%	41.2%
Tonbary,10 [92]	2000-8	Mansoura, Dakahlia, Lower Egypt	CS	Multi-transfused children	72	11.1%	N/A
El-Faramawy,12 [93]	N/A	Qena, Upper Egypt	CS	Multi-transfused children	33	39%	N/A
Abdelwahab,12 [94]	N/A	Cairo city, Cairo	CS	Multi-transfused children	100	40%	N/A
**Thalassemic patients**							
El-Nanawy,95 [19]	N/A	Alexandria city, Alexandria	CS	Children with thalassemia	18	44.4%	N/A
El Gohary,95 [36]	1990-2	Cairo city, Cairo	CS	Children with thalassemia	45	75.6%	N/A
Khalifa,04 [95]	2000-3	Cairo city, Cairo	CS	Children with thalassemia	56	69.6%	N/A
Abdalla,06 [96]	2005	Cairo city, Cairo / Banha, Qalubiya, Lower Egypt	CS	Children with thalassemia	33	60.6%	N/A
Omar,11 [97]	N/A	Cairo city, Cairo	CS		174	51.7%	32.2%
Mansour,12 [98]	2009-10	Mansoura, Dakahlia, Lower Egypt	CS	Children with thalassemia	200	19.5%	N/A
El-Faramawy,12 [93]	N/A	Qena, Upper Egypt	CS	Children with thalassemia	67	48%	N/A
**Schistosomiasis patients**							
Bassily,92 [99]	N/A	Nile River Delta, Lower Egypt	CS		31	41.9%	N/A
El-Nanawy,95 [19]	N/A	Alexandria city, Alexandria	CS		21	38.1%	N/A
El-Zayadi,97 [39]	N/A	N/A	CS		320	16.3%	N/A
Zekri,02 [100]	1998-00	Cairo city, Cairo	CS		47	42.5%	N/A
Arafa,05 [62]	2002-3	Zawiat Razin, Menoufia, Lower Egypt	CS	Schistomiasis patients treated with PAT	206	51.5%	N/A
El-Sabah,11 [101]	N/A	Rural area, Cairo/ Gharbia, Lower Egypt	CS	Schistosomiasis patients treated with PAT	50	84.0%	N/A
El-Sabah,11 [101]	N/A	Rural area, Cairo/ Gharbia, Lower Egypt	CS	Schistosomiasis patients treated orally up to 8 years ago	52	7.7%	N/A
**Hemodialysis patients**							
Hassan,93 [102]	N/A	N/A	CS		105	67.0%	N/A
Abdel-Wahab,94 [53]	1992	Cairo city, Cairo	CS		78	46.2%	N/A
El-Ahmady,94 [10]	N/A	Cairo city, Cairo	CS		25	84.0%	N/A
El Gohary,95 [36]	1990-2	Suez city, Suez and Ismailia, Lower Egypt	CS		108	70.4%	N/A
Gohar,95 [72]	N/A	N/A	CS		64	87.5%	N/A
Hassan,00 [103]	1996	Cairo city, Cairo	CS		210	59.0%	N/A
Shatat,00 [104]	1999	N/A	CS		83	78.5%	N/A
Gad,02 [105]	1998	Ismailia, Lower Egypt	CS		47	72.0%	N/A
Zekri,02 [100]	1998-00	Cairo city, Cairo	CS		30	56.6%	N/A
El Yazeed,06 [106]	2002-4	Cairo city, Cairo	CS		40	100.0%	N/A
Kandil,07 [22]	2004-6	Cairo city, Cairo	CS		31	51.6%	N/A
Hammad,09 [25]	2008	Mansoura, Dakahlia, Lower Egypt	CS		34	94.1%	N/A
Attia,10 [107]	2008-9	Cairo city, Cairo	CS		206	46.1%	N/A
Ibrahim,10 [108]	2007	Cairo city, Cairo	CS		100	75.0%	N/A
**Injection Drug Users (IDUs)**							
El-Ghazzawi,95 [109]	N/A	Alexandria city, Alexandria	CS		100	63.0%	N/A

*CS* convenience sampling, *N/A* not available, *PAT* parenteral antischistosomiasis therapy.

Among patients diagnosed with acute viral hepatitis, HCV prevalence ranged from as low as 4.3% [89] to as high as 78.7% [85]. Once more, we observed a higher prevalence among studies conducted in rural populations versus urban populations. A recent study conducted in 2010 reported an HCV prevalence of 8.7% among children with viral hepatitis [87].

The majority of studies in multi-transfused and thalassemia patients were conducted among children. High HCV prevalence rates were observed with averages of about 42% among multi-transfused children and about 58% among children with thalassemia. Multiple studies were also conducted among hemodialysis patients (mostly adults). Very high HCV prevalence was found in both adult populations and children on hemodialysis.

There were six studies that investigated HCV prevalence among schistosomiasis patients. Of these, only two explicitly mentioned previous PAT exposure. However, from the studies context, and given the high HCV prevalence across all of these studies, exposure to previous PAT campaigns seems to be implicitly assumed. Accordingly, we chose to classify these six studies in one subgroup: *schistosomiasis patients*, and not separate them into two categories based on previous PAT exposure.

Overall, the average HCV prevalence among schistosomiasis patients was about 38%. A recent study by Sabah *et al.* reported a prevalence of 84.0% among schistosomiasis patients treated with PAT 20 to 30 years ago, and a prevalence of 7.7% among schistosomiasis patients treated orally up to 8 years ago [101].

We were able to identify only one study conducted among IDUs in Egypt [109]. The study was conducted in Alexandria, among 100 IDUs, and HCV prevalence was reported to be 63.0% [109].

Figure 2B depicts the range of prevalence within each subgroup in studies conducted pre and post-2001. No distinct pattern can be discerned in the distribution of HCV prevalence pre- and post-2001 within each of the different subgroups.

### Prevalence of HCV among populations at indirect or intermediate risk of exposure

Populations at indirect or intermediate risk of exposure to HCV were classified into the following subcategories: diabetic patients, hospital outpatient attendees, hospitalized populations, household contacts of index cases (HCV positive cases), sexually transmitted infection (STI) patients, periodontal disease patients, prisoners, and populations working in select professions. Details are shown in Table S2, see Additional file 1.

Considerable HCV prevalence was reported among diabetic children in Egypt compared to other countries. A recent study conducted in 2010, among 692 diabetic children with an average age of 10.4 years, reported a prevalence of 2.5% [110]. Previous studies conducted among diabetic children reported much higher levels of 29.4% [19] and 44.1% [22]. In contrast, HCV prevalence among adult diabetic patients was 20.0% [100] and 60.3% [111]. Substantial HCV prevalence was also observed among patients attending hospitals, ranging between 0% [87] and 72.8% [112]. We identified one study conducted among periodontal disease patients reporting a prevalence of 13.0% [113]. Multiple studies were conducted investigating HCV prevalence among the children, spouses, and family contacts of HCV positive cases. Studies conducted among children of index cases usually focused on children of infected mothers to examine the vertical transmission of HCV. HCV RNA prevalence among infants born to HCV positive mothers ranged between 3.8% [28] and 25.0% [29]. HCV prevalence among spouses of index patients was as high as 35.5% [114]. In a study of family contacts of index patients, the prevalence was 5.7% [115].

Six studies were conducted among populations in select HCV-relevant professions (Table S2 of Additional file 1). HCV prevalence among health care workers averaged about 17%, whereas among barbers it was 12.3% [116]. We were able to identify only one study among prisoners which reported a prevalence of 31.4% [34].

**Figure 3:**
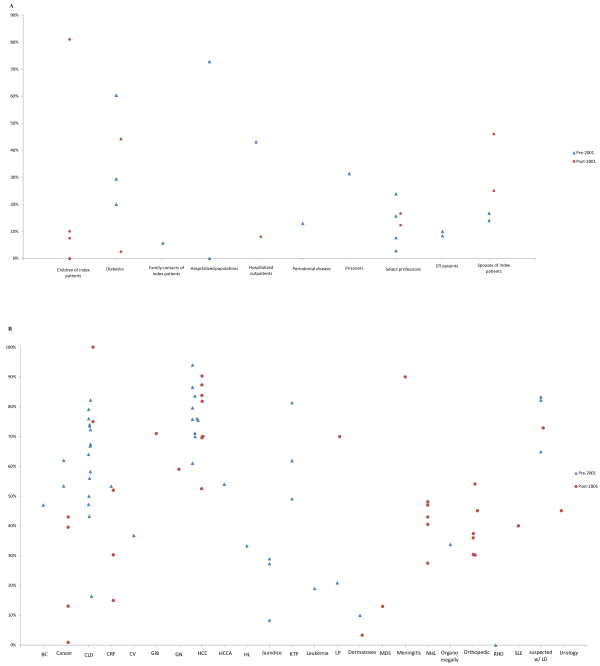
**Hepatitis C virus (HCV) prevalence among populations at indirect or intermediate risk and special clinical populations in Egypt, in studies conducted pre and post the 2001. A**: Graph depicting HCV prevalence among different populations at indirect or intermediate risk in Egypt. **B**: Graph depicting HCV prevalence among different special clinical populations^1^ in Egypt. In this figure, we included only stratified HCV prevalence measures, if these stratified measures were available. Otherwise, we included the overall prevalence measures in the study. ^1^Acronyms: BC: bladder cancer, CLD: chronic liver disease, CRF: chronic renal failure, CV: cutaneous vasculitis, GIB: gastrointestinal bleeding, GN: glomerulonephritis, HCC: hepatocellular carcinoma, HCCA:hilarcholangiocarcinoma, HL: Hodgkin’s lymphoma, KTP: kidney transplant patients, LD: liver diseae, LP:lichen planus, MDS: myelodysplastic syndrome NHL: non-Hodgkin’s lymphoma, RHD: rheumatic heart disease, SLE: systematic lupus erthymatosus.

The range of HCV prevalence within each subgroup in studies conducted pre- and post-2001 can be observed in Figure 3A. No distinct pattern can be discerned in the distribution of HCV prevalence pre- and post-2001 within each of the different subgroups.

### Prevalence of HCV among special clinical populations

A large fraction of studies were conducted among different clinical populations (Table S3 of Additional file 1). Overall, HCV prevalence was very high across all special clinical population groups. The average HCV prevalence among non-Hodgkin’s lymphoma (NHL) patients was roughly 41%, while among orthopedic patients it was about 39%. HCV prevalence among hepatocellular carcinoma (HCC) cases ranged between 61.0% and 90.3%, with a higher prevalence observed among rural versus urban populations [117]. Three studies were conducted among pediatric cancer patients [81,118,119]. HCV prevalence among children with leukemia was 19.0% [118]. HCV prevalence among patients with pediatric malignancies who had just ended chemotherapy was 39.6% [119]. More recently, Sharaf-Eldeen *et al.* reported HCV prevalence of 43.0% among children with malignant cancers [81].

No distinct pattern can be discerned in the distribution of HCV prevalence pre- and post-2001 within each of the different special clinical population subgroups (Figure 3B).

### RNA prevalence

Measures of RNA prevalence are included in Tables 2–3, and S2-S3 of Additional file 1. RNA prevalence was high across studies in the different population groups. Higher RNA prevalence was observed among studies conducted among high risk groups and special clinical populations compared to the general population and indirect or intermediate risk groups. Overall, the average RNA prevalence among those HCV-antibody positive was approximately 60%.

### Risk factors of HCV

Increasing age, a history of PAT, and residing in rural areas were by far the most common risk factors or associations with HCV infection across studies [26,62]. Other common risk factors were related to healthcare settings such as history of blood transfusions, invasive procedures, injections, perinatal care, and dental work [15,61,62,69,71,82,87]. Saleh *et al.* reported a greater risk for incident infection among women whose babies were delivered by a physician rather than by a nurse or a traditional birth attendant, in a health facility rather than at their home, and in women having complicated vaginal deliveries [15]. Among children, incident infection was associated with hospitalization and low birth weight [17]. Community and informal health provider related exposures such as circumcision, cautery, and injections were also associated with HCV infection [62,82,120]. A number of studies have also suggested intrafamilial transmission though the exact exposures responsible are not clear [14,121].

### Time trend analysis

#### General population model

Twenty six of the 87 general population studies had the year of data collection missing. The results of our paired t-test identified a mean difference of 3.1 years (95% CI: 2.6 to 3.6) between the year of publication and the year of data collection, for studies with both values present. We applied this time lag to estimate the year of data collection for studies missing this value.

In the univariate linear regression analyses for each subgroup separately, blood donors were the only subgroup showing a statistically significant change in HCV prevalence over time (p-value of 0.001). Table S4, see Additional file 1, reports the results of the univariate analyses.

**Figure 4:**
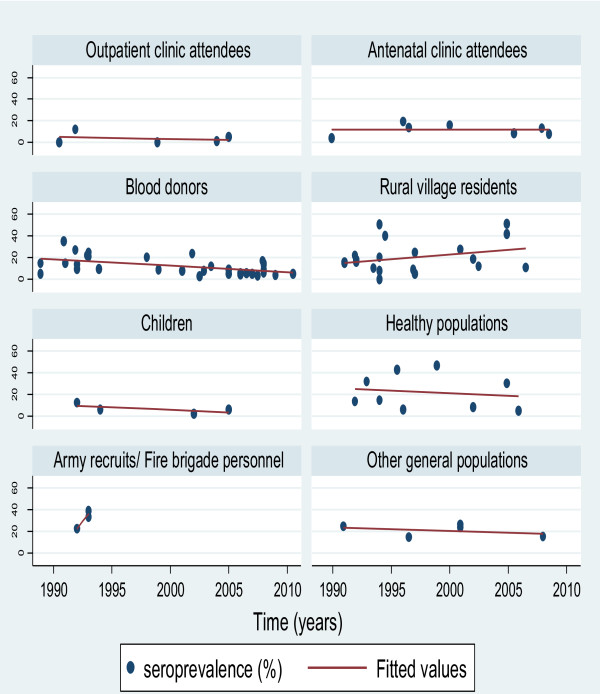
Time trend of hepatitis C prevalence among the different general population subgroups in Egypt.

In the multivariate linear regression analysis for the combined general population subgroups, there was no evidence of a statistically significant decline in HCV prevalence over time (p-value of 0.215). HCV prevalence in the general population changed at a rate of −0.24% per year (95% CI: -0.63 to 0.14). Figure 4 displays the trend in HCV prevalence with time in each of the eight general population subgroups.

#### Direct or high risk population model

Twenty five of the 46 direct or high risk population studies had the year of data collection missing. The results of the paired t-test identified a mean difference of 3.3 years (95% CI: 4.0 to 2.6) between the year of publication and the year of data collection. We applied this time lag to estimate the year of data collection for studies missing this value.

As was the case for the general population univariate and multivariate analyses, there was no evidence of a statistically significant decline in HCV prevalence over time for each high risk population subgroup (results not shown) and in the high risk population as a whole (p-value of 0.426). HCV prevalence in the high risk population declined at a rate of −0.38% per year (95% CI: -1.35 to 0.58). Figure S5, of Additional file 1, displays the trend in HCV prevalence with time in each of the five high risk subgroups. IDUs were excluded from this figure as there was only one observation.

## Discussion

We have systematically reviewed HCV infection prevalence and incidence across the different population groups in Egypt. The results of our study indicate that Egypt is enduring a large HCV disease burden, and is likely to be the most affected nation worldwide by this infection. HCV prevalence and incidence across the diverse population groups were found to be much higher than those in other countries around the globe [3,122,123]. This makes HCV and its complications one of the leading public health problems that Egypt has to confront today.

The results of this synthesis indicate that Egypt has endured a large HCV epidemic at the national level, and that there is substantial endemic HCV transmission in this country. HCV prevalence is at high levels across essentially all population groups, demonstrating the expansive nature of this epidemic, and that it is not isolated to specific population groups or geographic areas.

Despite being a major driver of HCV incidence and prevalence in many countries [124], the *relative* contribution of IDUs to HCV incidence and prevalence in Egypt is much smaller than that in other countries. This is because of the specific context of HCV in Egypt, where HCV transmission is associated with medical exposures in the context of a general population HCV epidemic. Still, there is a contribution from injecting drug use to HCV transmission that is, in terms of absolute scale, comparable to other countries. The prevalence of injecting drug use in Egypt is estimated to be 0.21% [125], and according to the only study we found, HCV prevalence among IDUs is 63% [109]. Considering that HCV prevalence in the population is 14.7% [1], injecting drug use may explain at most only about 1% of the national HCV prevalence in Egypt.

It is widely believed that the PAT campaigns to control schistosomiasis are the major drivers of the HCV epidemic in Egypt [5]. During the early twentieth century, schistosomiasis was highly prevalent in Egypt, especially in rural areas [6]. From the 1950s to the early 1980s, the Egyptian Ministry of Health led large-scale campaigns to control the disease [6]. Millions of people were treated with intravenous injections of tartar emetic, before an oral drug replaced this standard of care across the country in the 1980s [5]. Reuse of glass syringes and lax sterilization practices during PAT campaigns appear to have caused widespread infection with HCV, which by the 1990s, had replaced schistosomiasis as the primary cause of liver disease in Egypt [6,126].

Our study supports a major role for the PAT campaigns in driving HCV incidence. Different studies have shown a dramatic increase in HCV prevalence with age; a cohort effect that may be explained, at least in part, by the early association between PAT and HCV transmission [26,60-62,82,127]. Our results also highlight gender and geographical differences in HCV prevalence [36,64,71,75,128,129], with higher prevalence observed in males and rural dwellers compared to females and individuals living in urban areas. These differences may also be in part attributed to the PAT campaigns, as rural areas and males were more affected by the schistosomiasis disease burden and hence were main targets of these campaigns [5].

However, the totality of the evidence synthesized here suggests that the PAT campaigns are one driver among others of HCV transmission in Egypt, and that substantial HCV transmission is still ongoing. High HCV prevalence is found among hospitalized and the special clinical populations; populations that have experienced various facility-based medical procedures. Elevated HCV prevalence is also found among individuals exposed to even minor medical care procedures, within and beyond established health care facilities. Community studies have found strong correlations between HCV infection and different medical exposures such as injections, blood transfusions, surgical procedures, perinatal care, and dental treatment [15,61,62,69,71,82,87].

HCV prevalence among children, in particular, highlights the ongoing transmission of HCV in Egypt. Not only were these children born well after the end of the PAT campaigns, but also a large fraction of them were born after the implementation of the more stringent infection control measures in the country. Nevertheless, considerable prevalence levels are found among children in multiple studies. These studies suggest that children have been exposed to HCV vertically through mother-to-child transmission [24,25,27-29] (high RNA prevalence was documented among infants of HCV positive mothers, ranging between 3.8% and 11.1% [24,25,27-29,130,131]), or horizontally possibly through household exposures [14,114,115,121,131]. Medical exposures to HCV at a very young age have been also indicated [19,22,110,132]. High HCV levels were reported among thalassemic children [19,96,133], children on hemodialysis [22,132] and diabetic children [19,22,110].

HCV prevalence among the mothers of infected children, who tend to be young themselves, has been also associated with medical exposures and/or household exposures [24,25,27,28]. Injecting drug use is unlikely to contribute much to HCV prevalence among these mothers, given the context of the HCV epidemic in Egypt. Moreover, injecting drug use among women in the Middle East and North Africa region is believed to be marginal with only about 10% or less of IDUs being females in this region [134-136].

Results of our time trend analysis suggest that, contrary to expectations, there appears to be a small decline, though statistically not significant, in HCV prevalence over time in the general population and high risk population in Egypt. In the univariate subgroup-specific analyses, only blood donors have shown a statistically significant decline in HCV prevalence. However, this decline is difficult to interpret since recruitment of blood donors changed over time, particularly by excluding HCV positive individuals. While it can take a long time for the prevalence of HCV to decline after the PAT exposures, this fact may not be sufficient to explain the lack of tangible decline. Egypt’s population has almost doubled in the past two to three decades since the epidemic was first discovered, and well after the end of the PAT campaigns. The large influx of uninfected birth cohorts does not appear to have reduced HCV prevalence, possibly suggesting that HCV incidence has not declined as expected following the end of the PAT campaigns and adoption of more stringent infection control measures.

Another potential explanation for the lack of substantial decline in incidence is the very high baseline HCV prevalence in the country. For a given mode of transmission, such as reuse of unclean needles or syringes, the transmission risk is dependent on the probability that the needle/syringe was used previously on an HCV infected person, which is HCV RNA prevalence. Even if the prevalence of the modes of transmissions in Egypt today is similar to other neighboring countries, the high background prevalence can drive much more incidence. In neighboring Libya for example, with an HCV RNA prevalence of less than 1% [134,137], a reuse of an unclean needle is more than ten-fold less likely to lead to an HCV transmission than in Egypt where HCV RNA prevalence is 9.8% [1].

Our study identified the lack of an empirical nationally-representative incidence study. It is a priority to document current HCV incidence rate in the population, which continues to be the most critical open question in HCV epidemiology in Egypt today, and is subject to much debate [127,138]. A recent mathematical modeling study, based on the EDHS data, estimated that the average HCV incidence rate over the lifetime of the living Egyptian population cohort to be 6.9 per 1,000 person-year [7]. This estimate however does not capture the temporal trend in HCV incidence rate and may not be representative of the current level of HCV transmission. A recent study suggested that current HCV incidence rate is about 2.0 per 1000 person-year [127].

While the evidence for an epidemic at the national level is overwhelming, some of the potential drivers of this epidemic, such as the PAT campaigns and contaminated blood, are no longer present. Therefore, it seems plausible that HCV incidence rate has declined drastically in the last two decades since the discovery of the epidemic in 1991–1992 [30,139], as Breban *et al.* have recently suggested through their incidence estimate [127]. Nevertheless, our study could not identify a signature for a major reduction in incidence, and the totality of evidence points towards substantial ongoing HCV transmission, though the precise scale of which is not yet known.

In terms of limitations, there was an element of subjectivity in classifying different studies into different population subgroups. For example, studies conducted in rural children were classified under children even though they fall into two subgroups: rural populations and children. Furthermore, there was variability in the diagnostic assays used across the studies. Earlier studies typically reported the use of 1st and 2nd generation ELISA tests, which lack the sensitivity and specificity of the 3rd generation ELISA tests. Such variability in assays may impact the representativeness of the reported prevalence measures. There was also considerable variability in methodology and quality among the studies assessed. Most of the studies identified were cross-sectional or case–control in study design. Sample size varied widely across studies. The sampling was most often convenience sampling, though several studies have used probability-based samples [1,61,69], most notably the EDHS conducted in 2008 [1]. For a fraction of the studies the year of data collection was not available but was estimated using the year of publication, this may affect the time trend analysis. Given that only statistically significant risk factors were extracted, this may have introduced a positive-association selection bias in our reporting of risk factors.

## Conclusions

Our study highlights that Egypt is confronted with an HCV disease burden of historical proportions. An HCV epidemic at the national level must have occurred with substantial transmission still ongoing today. As opposed to other countries where HCV dynamics is focused in specific high risk groups, such as IDUs, HCV transmission in Egypt has reached diverse population groups including those not conventionally identified to be at risk of infection. HCV transmission appears to be focused in formal and informal health care settings, though transmission may be occurring in the community and at the household level, but through poorly-identified exposures.

HCV prevention in Egypt must be a national priority. Policymakers and public health and medical care stakeholders need to introduce and implement further prevention measures targeting the iatrogenic transmission routes, such as very stringent infection control practices. Scientific research needs to be expanded to measure current HCV incidence rate and to identify precisely the modes of HCV transmission in medical care, community, and household settings. Such studies will pave the way for effective prevention interventions that can be developed, experimentally tested, and implemented. It is also essential to develop cost-effective strategies for treatment and case management of the large pool of 5–7 million chronically infected persons with HCV in Egypt.

## Abbreviations

HCV: Hepatitis C virus; EDHS: Egyptian demographic health survey; PAT: Parenteral antischistosomiasis therapy; IDU: Injecting drugs use; STI: Sexually transmitted infection; NHL: Non-Hodgkin’s lymphoma; HCC: Hepatocellular carcinoma; PBS: Probability-based sampling; CS: Convenience sampling; HCCA: Hilarcholangiocarcinoma; KTP: Kidney transplant patient; LP: Lichen planus; HL: Hodgkin’s lymphoma; CV: Cutaneous vasculitis; CLD: Chronic liver disease; BC: Bladder cancer; Rheumatic HD: Rheumatic heart disease; CRF: Chronic renal failure; LD: Liver disease; GIB: Gastrointestinal bleeding; SLE: Systemic lupus erythematosus; MDS: Myelodysplastic syndrome; GN: Glomerulonephritis.

## Competing interests

The authors declare that they have no competing interests.

## Authors’ contributions

YM and SR conducted the literature review and data retrieval. YM conducted extraction, analysis and wrote the first draft of the paper. GM participated in study design. FDM contributed to the study design and analyses. LJA conceived and led the design of the study, analyses, and drafting of the article. All authors contributed to discussion of the results and writing of the manuscript. All authors read and approved the final manuscript.

## Pre-publication history

The pre-publication history for this paper can be accessed here:

http://www.biomedcentral.com/1471-2334/13/288/prepub

## Supplementary Material

Additional file 1: Table S1 PRISMA Checklist. **Table S2.** Prevalence of hepatitis C virus among populations at indirect or intermediate risk of exposure in Egypt. **Table S3.** Prevalence of hepatitis C virus among special clinical populations in Egypt. **Table S4.** Hepatitis C virus time trend analysis for each general population subgroup. **Figure S5.** Hepatitis C virus time trend analysis among populations at direct or high risk of exposure. Click here for file
