# Supplementary material for: The epidemiology of hepatitis C virus in Egypt: a systematic review and data synthesis
Source: BMC Infect Dis. 2013 Jun 24;13:288. doi: 10.1186/1471-2334-13-288 (PMC3702438; doi:10.1186/1471-2334-13-288)
Supplement: Additional file 1: Table S1 — PRISMA Checklist. Table S2. Prevalence of hepatitis C virus among populations at indirect or intermediate risk of exposure in Egypt. Table S3. Prevalence of hepatitis C virus among special clinical populations in Egypt. Table S4. Hepatitis C virus time trend analysis for each general population subgroup. Figure S5. Hepatitis C virus time trend analysis among populations at direct or high risk of exposure. [file 1471-2334-13-288-S1.docx]

Supplement

**Content**

**Table S1-** PRISMA Checklist

**Table S2-** Prevalence of hepatitis C virus among populations at indirect or intermediate risk of exposure in Egypt

**Table S3-** Prevalence of hepatitis C virus among special clinical populations in Egypt

**Table S4**- Hepatitis C virus time trend analysis for each general population subgroup

**Figure S5-** Hepatitis C virus time trend analysis among populations at direct or high risk of exposure

**Table S1-** PRISMA Checklist


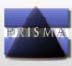
**PRISMA 2009 Checklist**

| **Section/topic** | **#** | **Checklist item** | **Reported on page #** |
| --- | --- | --- | --- |
| **TITLE** | | |  |
| Title | 1 | Identify the report as a systematic review, meta-analysis, or both. | 1 |
| **ABSTRACT** | | |  |
| Structured summary | 2 | Provide a structured summary including, as applicable: background; objectives; data sources; study eligibility criteria, participants, and interventions; study appraisal and synthesis methods; results; limitations; conclusions and implications of key findings; systematic review registration number. | 2 |
| **INTRODUCTION** | | |  |
| Rationale | 3 | Describe the rationale for the review in the context of what is already known. | 4 |
| Objectives | 4 | Provide an explicit statement of questions being addressed with reference to participants, interventions, comparisons, outcomes, and study design (PICOS). | 4 |
| **METHODS** | | |  |
| Protocol and registration | 5 | Indicate if a review protocol exists, if and where it can be accessed (e.g., Web address), and, if available, provide registration information including registration number. | 5 |
| Eligibility criteria | 6 | Specify study characteristics (e.g., PICOS, length of follow-up) and report characteristics (e.g., years considered, language, publication status) used as criteria for eligibility, giving rationale. | 5-6 |
| Information sources | 7 | Describe all information sources (e.g., databases with dates of coverage, contact with study authors to identify additional studies) in the search and date last searched. | 5-6 |
| Search | 8 | Present full electronic search strategy for at least one database, including any limits used, such that it could be repeated. | 35 |
| Study selection | 9 | State the process for selecting studies (i.e., screening, eligibility, included in systematic review, and, if applicable, included in the meta-analysis). | 5-6 |
| Data collection process | 10 | Describe method of data extraction from reports (e.g., piloted forms, independently, in duplicate) and any processes for obtaining and confirming data from investigators. | 6-7 |
| Data items | 11 | List and define all variables for which data were sought (e.g., PICOS, funding sources) and any assumptions and simplifications made. | 6-7 |
| Risk of bias in individual studies | 12 | Describe methods used for assessing risk of bias of individual studies (including specification of whether this was done at the study or outcome level), and how this information is to be used in any data synthesis. | - |
| Summary measures | 13 | State the principal summary measures (e.g., risk ratio, difference in means). | 6 |
| Synthesis of results | 14 | Describe the methods of handling data and combining results of studies, if done, including measures of consistency (e.g., I^2^) for each meta-analysis. | - |

| **Section/topic** | **#** | **Checklist item** | **Reported on page #** |
| --- | --- | --- | --- |
| Risk of bias across studies | 15 | Specify any assessment of risk of bias that may affect the cumulative evidence (e.g., publication bias, selective reporting within studies). | - |
| Additional analyses | 16 | Describe methods of additional analyses (e.g., sensitivity or subgroup analyses, meta-regression), if done, indicating which were pre-specified. | 8-9 |
| **RESULTS** | | |  |
| Study selection | 17 | Give numbers of studies screened, assessed for eligibility, and included in the review, with reasons for exclusions at each stage, ideally with a flow diagram. | 36 |
| Study characteristics | 18 | For each study, present characteristics for which data were extracted (e.g., study size, PICOS, follow-up period) and provide the citations. | 24-34  SA: 4-12 |
| Risk of bias within studies | 19 | Present data on risk of bias of each study and, if available, any outcome level assessment (see item 12). | - |
| Results of individual studies | 20 | For all outcomes considered (benefits or harms), present, for each study: (a) simple summary data for each intervention group (b) effect estimates and confidence intervals, ideally with a forest plot. | 24-34 |
| Synthesis of results | 21 | Present results of each meta-analysis done, including confidence intervals and measures of consistency. | - |
| Risk of bias across studies | 22 | Present results of any assessment of risk of bias across studies (see Item 15). | - |
| Additional analysis | 23 | Give results of additional analyses, if done (e.g., sensitivity or subgroup analyses, meta-regression [see Item 16]). | 16-17 |
| **DISCUSSION** | | |  |
| Summary of evidence | 24 | Summarize the main findings including the strength of evidence for each main outcome; consider their relevance to key groups (e.g., healthcare providers, users, and policy makers). | 17-21 |
| Limitations | 25 | Discuss limitations at study and outcome level (e.g., risk of bias), and at review-level (e.g., incomplete retrieval of identified research, reporting bias). | 20-21 |
| Conclusions | 26 | Provide a general interpretation of the results in the context of other evidence, and implications for future research. | 22 |
| **FUNDING** | | |  |
| Funding | 27 | Describe sources of funding for the systematic review and other support (e.g., supply of data); role of funders for the systematic review. | 3 |

*From:*  Moher D, Liberati A, Tetzlaff J, Altman DG, The PRISMA Group (2009). Preferred Reporting Items for Systematic Reviews and Meta-Analyses: The PRISMA Statement. PLoS Med 6(6): e1000097. doi:10.1371/journal.pmed1000097

**Table S2.** Studies reporting prevalence of hepatitis C virus among populations at indirect or intermediate risk of exposure in Egypt.

| **Citation** | **Year** | **Location** | **Sampling** | **Population characteristic** | **Sample size** | **Sero-prevalence** | **RNA prevalence** |
| --- | --- | --- | --- | --- | --- | --- | --- |
|  |  |  |  |  |  |  |  |
| **Diabetic patients** | | | | | | | |
| [El-Nanawy,95](file:///C:\Users\Risk%20groups%20tables.xlsx#RANGE!_ENREF_54)[[1](#_ENREF_1)] | N/A | Alexandria city, Alexandria | CS | Children | 17 | 29.4% | N/A |
| [Zekri,02](file:///C:\Users\Risk%20groups%20tables.xlsx#RANGE!_ENREF_128)[[2](#_ENREF_2)] | 1998-00 | Cairo city, Cairo | CS |  | 30 | 20.0% | N/A |
| [Kandil,07](file:///C:\Users\Risk%20groups%20tables.xlsx#RANGE!_ENREF_119)[[3](#_ENREF_3)] | 2004-6 | Cairo city, Cairo | CS | Children | 34 | 44.1% | N/A |
| Elmagd,08[[4](#_ENREF_4)] | 1976-04 | Mansoura, Dakahlia, Lower Egypt | CS |  | 286 | 60.30% | N/A |
| [El-Karaksy,10](file:///C:\Users\Risk%20groups%20tables.xlsx#RANGE!_ENREF_1)[[5](#_ENREF_5)] | 2007-8 | Cairo city, Cairo | CS | Children | 692 | 2.5% | N/A |
|  |  |  |  |  |  |  |  |
| **Hospitalized outpatients** | | | | | | | |
| Halim,99[[6](#_ENREF_6)] | 1996 | Cairo city, Cairo | CS |  | 51 | 43.1% | N/A |
| [Kalil,10](file:///C:\Users\Risk%20groups%20tables.xlsx#RANGE!_ENREF_5)[[6](#_ENREF_6)] | 2004-5 | Assuit, Upper Egypt | CS | Children | 150 | 8.0% | 4.7% |
|  |  |  |  |  |  |  |  |
| **Hospitalized populations** | | | | | | | |
| [Khalifa,93](file:///C:\Users\Risk%20groups%20tables.xlsx#RANGE!_ENREF_65)[[7](#_ENREF_7)] | 1990-1 | Cairo city, Cairo | CS | Hospitalized children | 84 | 0.0% | N/A |
| [El-Medany,99](file:///C:\Users\Risk%20groups%20tables.xlsx#RANGE!_ENREF_41)[[8](#_ENREF_8)] | N/A | Mansoura, Dakahlia, Lower Egypt | CS | Surgery patients | 44 | 72.8% | N/A |
|  |  |  |  |  |  |  |  |
| **Children of index cases** | | | | | | | |
| [Agha,98](file:///C:\Users\Risk%20groups%20tables.xlsx#RANGE!_ENREF_44)[[9](#_ENREF_9)] | 1996-7 | Mansoura, Dakahlia, Lower Egypt | CS | Newborns to HCV+ mothers | 18 | N/A | 11.1% |
| [Madwar,99](file:///C:\Users\Risk%20groups%20tables.xlsx#RANGE!_ENREF_23)[[76](#_ENREF_76)] | N/A | N/A | CS | Children of chronic HCV patients | 355 | 0.0% | N/A |
| [Kassem,00](file:///C:\Users\Risk%20groups%20tables.xlsx#RANGE!_ENREF_38)[[10](#_ENREF_10)] | 1996 | Alexandria city, Alexandria | CS | Infants of HCV+ mothers | 19 | N/A | 9.0% |
| [Shebl,09](file:///C:\Users\Risk%20groups%20tables.xlsx#RANGE!_ENREF_11)[[11](#_ENREF_11)] | 1997-01 | Nile River Delta, Lower Egypt | CS | Infants to HCV RNA positive mothers | 232 | N/A | 6.5% |
| [Zahran,10](file:///C:\Users\Risk%20groups%20tables.xlsx#RANGE!_ENREF_2)[[12](#_ENREF_12)] | 2008-9 | Assuit, Upper Egypt | CS | Children of HCV+ mothers, at birth | 40 | 0% | 5.0% |
| [Zahran,10](file:///C:\Users\Risk%20groups%20tables.xlsx#RANGE!_ENREF_2)[[12](#_ENREF_12)] | 2008-9 | Assuit, Upper Egypt | CS | Children of HCV+ mothers at 3 months | 40 | 7.5% | 7.5% |
| [Zahran,10](file:///C:\Users\Risk%20groups%20tables.xlsx#RANGE!_ENREF_2)[[12](#_ENREF_12)] | 2008-9 | Assuit, Upper Egypt | CS | Children of HCV+ mothers at 8 months | 40 | 10.0% | 10.0% |
| [Abdulqawi,10](file:///C:\Users\Risk%20groups%20tables.xlsx#RANGE!_ENREF_6)[[13](#_ENREF_13)] | 2003-8 | Benha, Qalubiya, Lower Egypt | CS | Infants of infected women, 1^st^ month of life | 53 | 81% | 13.0% |
| [Abdulqawi,10](file:///C:\Users\Risk%20groups%20tables.xlsx#RANGE!_ENREF_6)[[13](#_ENREF_13)] | 2003-8 | Benha, Qalubiya, Lower Egypt | CS | Infants of infected women, 6^th^ month of life | 53 | N/A | 3.8% |
| Abo [Elmagd,11](file:///C:\Users\Risk%20groups%20tables.xlsx#RANGE!_ENREF_110)[[14](#_ENREF_14)] | N/A | N/A | CS | Infants of HCV infected mothers | 8 | N/A | 25.0% |
|  |  |  |  |  |  |  |  |
| **Spouses of index patients** | | | | | | | |
| [El-Zayadi,97](file:///C:\Users\Risk%20groups%20tables.xlsx#RANGE!_ENREF_48)[[15](#_ENREF_15)] | N/A | Cairo city, Cairo | CS |  | N/A | 16.7% | N/A |
| [Madwar,99](file:///C:\Users\Risk%20groups%20tables.xlsx#RANGE!_ENREF_23)[[76](#_ENREF_76)] | N/A | N/A | CS |  | 200 | 14.0% | N/A |
| [Morad,11](file:///C:\Users\Risk%20groups%20tables.xlsx#RANGE!_ENREF_122)[[16](#_ENREF_16)] | N/A | N/A | CS | Males | 100 | 25.0% | N/A |
| [Morad,11](file:///C:\Users\Risk%20groups%20tables.xlsx#RANGE!_ENREF_122)[[16](#_ENREF_16)] | N/A | N/A | CS | Females | 100 | 46.0% | N/A |
| [Morad,11](file:///C:\Users\Risk%20groups%20tables.xlsx#RANGE!_ENREF_122)[[16](#_ENREF_16)] | N/A | N/A | CS |  | 200 | 35.5% | N/A |
|  |  |  |  |  |  |  |  |
| **Family contacts of index patients** | | | | | | | |
| [El-Zayadi,97](file:///C:\Users\Risk%20groups%20tables.xlsx#RANGE!_ENREF_48)[[15](#_ENREF_15)] | N/A | Cairo city, Cairo | CS |  | 265 | 5.7% | 1.1% |
|  |  |  |  |  |  |  |  |
| **STI patients** | | | | | | | |
| [Hassan,93](file:///C:\Users\Risk%20groups%20tables.xlsx#RANGE!_ENREF_63)[[17](#_ENREF_17)] | N/A | N/A | CS |  | 83 | 10.0% | N/A |
| [Ali,98](file:///C:\Users\Risk%20groups%20tables.xlsx#RANGE!_ENREF_22)[[18](#_ENREF_18)] | 1993-5 | N/A | CS |  | 95 | 8.4% | N/A |
|  |  |  |  |  |  |  |  |
| **Prisoners** | | | | | | | |
| [Quinti,95](file:///C:\Users\Risk%20groups%20tables.xlsx#RANGE!_ENREF_53)[[19](#_ENREF_19)] | 1992-4 | Alexandria city, Alexandria | CS |  | 124 | 31.4% | N/A |
|  |  |  |  |  |  |  |  |
| **Select professions** | | | | | | | |
| [Shalaby,10](file:///C:\Users\Risk%20groups%20tables.xlsx#RANGE!_ENREF_4)[[20](#_ENREF_20)] | 2007 | Gharbia, Lower Egypt | CS | Barbers | 308 | 12.3% | 9.1% |
| [Hindy,95](file:///C:\Users\Risk%20groups%20tables.xlsx#RANGE!_ENREF_15)[[21](#_ENREF_21)] | N/A | Cairo city, Cairo | CS | Dentists | 35 | 2.9% | N/A |
| [El-Ahmady,94](file:///C:\Users\Risk%20groups%20tables.xlsx#RANGE!_ENREF_62)[[22](#_ENREF_22)] | N/A | Cairo city, Cairo | CS | Healthcare workers | 159 | 23.9% | N/A |
| [El Gohary,95](file:///C:\Users\Risk%20groups%20tables.xlsx#RANGE!_ENREF_57)[[66](#_ENREF_66)] | 1990-2 | Suez city, Suez and Ismailia, Lower Egypt | CS | Healthcare workers | 78 | 7.7% | N/A |
| [Yates,99](file:///C:\Users\Risk%20groups%20tables.xlsx#RANGE!_ENREF_43)[[23](#_ENREF_23)] | N/A | Cairo city, Cairo | CS | Healthcare workers | 466 | 15.7% | N/A |
| [Abdelwahab,11](file:///C:\Users\Risk%20groups%20tables.xlsx#RANGE!_ENREF_91)[[24](#_ENREF_24)] | 2008-10 | Menoufia, Lower Egypt | CS | Healthcare workers | 842 | 16.6% | 12.0% |
|  |  |  |  |  |  |  |  |
| **Periodontal disease patients** | | | | | | | |
| [Farghaly,98](file:///C:\Users\Risk%20groups%20tables.xlsx#RANGE!_ENREF_24)[[25](#_ENREF_25)] | N/A | N/A | CS |  | 100 | 13.0% | N/A |
| CS = convenience sampling; N/A = not available | | | | | | | |

**Table S3.** Studies reporting hepatitis C virus prevalence among special clinical populations in Egypt.

| **Citation** | **Year** | **Location** | **Sampling** | **Population characteristic** | **Sample size** | **Sero-prevalence** | **RNA prevalence** |
| --- | --- | --- | --- | --- | --- | --- | --- |
|  |  |  |  |  |  |  |  |
| **non-Hodgkin’s Lymphoma (NHL) patients** | | | | | | | |
| [Cowgill,04](file:///D:\Laith\Files\Pipeline\HCV\Yousra\Paper%20tables.xlsx#RANGE!_ENREF_88)[[26](#_ENREF_26)] | 1999-2003 | Cairo city, Cairo | CS |  | 220 | 48.1% | 42.7% |
| [El-Sayed,06](file:///D:\Laith\Files\Pipeline\HCV\Yousra\Paper%20tables.xlsx#RANGE!_ENREF_80)[[27](#_ENREF_27)] | 2002 | Cairo city, Cairo | CS |  | 29 | 27.5% | 20.7% |
| [Goldman,09](file:///D:\Laith\Files\Pipeline\HCV\Yousra\Paper%20tables.xlsx#RANGE!_ENREF_12)[[28](#_ENREF_28)] | 1999-2004 | Cairo city, Cairo | CS |  | 296 | 47.0% | 38.9% |
| [Kassem,09](file:///D:\Laith\Files\Pipeline\HCV\Yousra\Paper%20tables.xlsx#RANGE!_ENREF_120)[[29](#_ENREF_29)] | 2008-09 | N/A | CS |  | 37 | 40.5% | N/A |
| Farawela,12[[30](#_ENREF_30)] | 2010-1 | Cairo city, Cairo | CS |  | 100 | 43% | N/A |
|  |  |  |  |  |  |  |  |
| **Orthopedic patients** | | | | | | | |
| [Cowgill,04](file:///D:\Laith\Files\Pipeline\HCV\Yousra\Paper%20tables.xlsx#RANGE!_ENREF_88)[[26](#_ENREF_26)] | 1999-2003 | Cairo city, Cairo | CS |  | 222 | 36.0% | 23.4% |
| [Ezzat,05](file:///D:\Laith\Files\Pipeline\HCV\Yousra\Paper%20tables.xlsx#RANGE!_ENREF_107)[[31](#_ENREF_31)] | N/A | Cairo city, Cairo | CS | Orthopedic patients : urban males | 63 | 30.2% | N/A |
| [Ezzat,05](file:///D:\Laith\Files\Pipeline\HCV\Yousra\Paper%20tables.xlsx#RANGE!_ENREF_107)[[31](#_ENREF_31)] | N/A | Cairo city, Cairo | CS | Orthopedic patients: urban females | 23 | 30.4% | N/A |
| [Ezzat,05](file:///D:\Laith\Files\Pipeline\HCV\Yousra\Paper%20tables.xlsx#RANGE!_ENREF_107)[[31](#_ENREF_31)] | N/A | Cairo city, Cairo | CS | Orthopedic patients : rural males | 113 | 45.1% | N/A |
| [Ezzat,05](file:///D:\Laith\Files\Pipeline\HCV\Yousra\Paper%20tables.xlsx#RANGE!_ENREF_107)[[31](#_ENREF_31)] | N/A | Cairo city, Cairo | CS | Orthopedic patients: rural females | 37 | 54.1% | N/A |
| [Goldman,09](file:///D:\Laith\Files\Pipeline\HCV\Yousra\Paper%20tables.xlsx#RANGE!_ENREF_12)[[28](#_ENREF_28)] | 1999-2004 | Cairo city, Cairo | CS |  | 786 | 37.4% | 23.8% |
|  |  |  |  |  |  |  |  |
| **Hilar cholangiocarcinoma patients** | | | | | | | |
| [Abdel Wahab, 07](file:///D:\Laith\Files\Pipeline\HCV\Yousra\Paper%20tables.xlsx#RANGE!_ENREF_14)[[32](#_ENREF_32)] | 1995-04 | Mansoura, Dakahlia, Lower Egypt | CS |  | 440 | 54.0% | N/A |
|  |  |  |  |  |  |  |  |
| **Kidney transplant patients** | | | | | | | |
| [Gohar,95](file:///D:\Laith\Files\Pipeline\HCV\Yousra\Paper%20tables.xlsx#RANGE!_ENREF_58)[[33](#_ENREF_33)] | N/A | N/A | CS |  | 16 | 81.3% | N/A |
| [Sabry,07](file:///D:\Laith\Files\Pipeline\HCV\Yousra\Paper%20tables.xlsx#RANGE!_ENREF_15)[[34](#_ENREF_34)] | 1993-96 | Mansoura, Dakahlia, Lower Egypt | CS |  | 273 | 61.9% | N/A |
| Abu Elmagd,08[[4](#_ENREF_4)] | 1976-2004 | Mansoura, Dakahlia, Lower Egypt | CS |  | 316 | 49.1% | N/A |
|  |  |  |  |  |  |  |  |
| **Lichen Planus patients** | | | | | | | |
| [Ibrahim,99](file:///D:\Laith\Files\Pipeline\HCV\Yousra\Paper%20tables.xlsx#RANGE!_ENREF_40)[[35](#_ENREF_35)] | 1996-97 | Alexandria city, Alexandria | CS |  | 43 | 20.9% | N/A |
| [Amer,07](file:///D:\Laith\Files\Pipeline\HCV\Yousra\Paper%20tables.xlsx#RANGE!_ENREF_16)[[36](#_ENREF_36)] | N/A | N/A | CS |  | 30 | 70.0% | N/A |
|  |  |  |  |  |  |  |  |
| **Patients with dermotoses** | | | | | | | |
| [Ibrahim,99](file:///D:\Laith\Files\Pipeline\HCV\Yousra\Paper%20tables.xlsx#RANGE!_ENREF_40)[[35](#_ENREF_35)] | 1996-97 | Alexandria city, Alexandria | CS |  | 30 | 10.0% | N/A |
| [Amer,07](file:///D:\Laith\Files\Pipeline\HCV\Yousra\Paper%20tables.xlsx#RANGE!_ENREF_16)[[36](#_ENREF_36)] | N/A | N/A | CS |  | 30 | 3.3% | N/A |
|  |  |  |  |  |  |  |  |
| **Hodgkin’s Lymphoma patients** | | | | | | | |
| [Zekri,02](file:///D:\Laith\Files\Pipeline\HCV\Yousra\Paper%20tables.xlsx#RANGE!_ENREF_128)[[37](#_ENREF_37)] | 1998-2000 | Cairo city, Cairo | CS |  | 30 | 33.3% | N/A |
|  |  |  |  |  |  |  |  |
| **Hepatocellular carcinoma patients** | | | | | | | |
| [Darwish,93](file:///D:\Laith\Files\Pipeline\HCV\Yousra\Paper%20tables.xlsx#RANGE!_ENREF_71)[[38](#_ENREF_38)] | N/A | Cairo city, Cairo | CS |  | 70 | 70.0% | N/A |
| [Mabrouk,97](file:///D:\Laith\Files\Pipeline\HCV\Yousra\Paper%20tables.xlsx#RANGE!_ENREF_45)[[39](#_ENREF_39)] | 1995-96 | N/A | CS |  | 32 | 94.0% | 28% |
| [Darwish,97](file:///D:\Laith\Files\Pipeline\HCV\Yousra\Paper%20tables.xlsx#RANGE!_ENREF_49)[[40](#_ENREF_40)] | N/A | N/A | CS |  | 94 | 75.5% | N/A |
| Khalifa,99[[41](#_ENREF_41)] | N/A | N/A | CS |  | 61 | 83.6% | N/A |
| [Yates,99](file:///D:\Laith\Files\Pipeline\HCV\Yousra\Paper%20tables.xlsx#RANGE!_ENREF_43)[[23](#_ENREF_23)] | N/A | Cairo city, Cairo | CS |  | 131 | 76.0% | N/A |
| [Abdel-Wahab,00](file:///D:\Laith\Files\Pipeline\HCV\Yousra\Paper%20tables.xlsx#RANGE!_ENREF_104)[[42](#_ENREF_42)] | 1994-99 | Mansoura, Dakahlia, Lower Egypt | CS |  | 385 | 61.0% | N/A |
| [Hassan,01](file:///D:\Laith\Files\Pipeline\HCV\Yousra\Paper%20tables.xlsx#RANGE!_ENREF_33)[[43](#_ENREF_43)] | 1995-96 | Cairo city, Cairo | CS |  | 33 | 75.8% | N/A |
| [Rahman El-Zayadi,01](file:///D:\Laith\Files\Pipeline\HCV\Yousra\Paper%20tables.xlsx#RANGE!_ENREF_124)[[44](#_ENREF_44)] | 1992-95 | Cairo city, Cairo | CS |  | 200 | 71.1% | N/A |
| [Zekri,02](file:///D:\Laith\Files\Pipeline\HCV\Yousra\Paper%20tables.xlsx#RANGE!_ENREF_128)[[37](#_ENREF_37)] | 1998-2000 | Cairo city, Cairo | CS |  | 37 | 86.5% | N/A |
| [Ezzat,05](file:///D:\Laith\Files\Pipeline\HCV\Yousra\Paper%20tables.xlsx#RANGE!_ENREF_107)[[31](#_ENREF_31)] | N/A | Cairo city, Cairo | CS | HCC patients: urban males | 63 | 87.3% | N/A |
| [Ezzat,05](file:///D:\Laith\Files\Pipeline\HCV\Yousra\Paper%20tables.xlsx#RANGE!_ENREF_107)[[31](#_ENREF_31)] | N/A | Cairo city, Cairo | CS | HCC patients: urban females | 23 | 69.6% | N/A |
| [Ezzat,05](file:///D:\Laith\Files\Pipeline\HCV\Yousra\Paper%20tables.xlsx#RANGE!_ENREF_107)[[31](#_ENREF_31)] | N/A | Cairo city, Cairo | CS | HCC patients: rural males | 113 | 90.3% | N/A |
| [Ezzat,05](file:///D:\Laith\Files\Pipeline\HCV\Yousra\Paper%20tables.xlsx#RANGE!_ENREF_107)[[31](#_ENREF_31)] | N/A | Cairo city, Cairo | CS | HCC patients: rural females | 37 | 83.8% | N/A |
| [Abdel-Wahab,07](file:///D:\Laith\Files\Pipeline\HCV\Yousra\Paper%20tables.xlsx#RANGE!_ENREF_75)[[45](#_ENREF_45)] | 1992-2005 | Mansoura, Dakahlia, Lower Egypt | CS |  | 1,012 | 79.6% | N/A |
| [Abdel-Wahab,08](file:///D:\Laith\Files\Pipeline\HCV\Yousra\Paper%20tables.xlsx#RANGE!_ENREF_97)[[46](#_ENREF_46)] | 2005-06 | Mansoura, Dakahlia, Lower Egypt | CS |  | 80 | 70.0% | N/A |
| [El Bassuoni,08](file:///D:\Laith\Files\Pipeline\HCV\Yousra\Paper%20tables.xlsx#RANGE!_ENREF_114)[[47](#_ENREF_47)] | N/A | N/A | CS |  | 15 | 81.8% | N/A |
| [Abdel-Maksoud,09](file:///D:\Laith\Files\Pipeline\HCV\Yousra\Paper%20tables.xlsx#RANGE!_ENREF_109)[[48](#_ENREF_48)] | N/A | N/A | CS |  | 40 | 52.5% | N/A |
| Taha,12[[49](#_ENREF_49)] | 2007 | N/A | CS |  | 1,643 | 70% | N/A |
|  |  |  |  |  |  |  |  |
| **Leukemia patients** | | | | | | | |
| [Meir,01](file:///D:\Laith\Files\Pipeline\HCV\Yousra\Paper%20tables.xlsx#RANGE!_ENREF_36)[[50](#_ENREF_50)] | N/A | Cairo city, Cairo | CS | Children with leukemia | 54 | 19.0% | N/A |
|  |  |  |  |  |  |  |  |
| **Cutaneous vasculitis patients** | | | | | | | |
| [Ibrahim,99](file:///D:\Laith\Files\Pipeline\HCV\Yousra\Paper%20tables.xlsx#RANGE!_ENREF_40)[[35](#_ENREF_35)] | 1996-97 | Alexandria city, Alexandria | CS |  | 19 | 36.8% | N/A |
|  |  |  |  |  |  |  |  |
| **Chronic Liver Disease patients** | | | | | | | |
| [El-Zayadi,92](file:///D:\Laith\Files\Pipeline\HCV\Yousra\Paper%20tables.xlsx#RANGE!_ENREF_69)[[51](#_ENREF_51)] | N/A | N/A | CS | Patients diagnosed with non-A non-B Hepatitis related CLD | 160 | 66.8% | N/A |
| [Abdel-Wahab,94](file:///D:\Laith\Files\Pipeline\HCV\Yousra\Paper%20tables.xlsx#RANGE!_ENREF_60)[[52](#_ENREF_52)] | 1992 | Cairo city, Cairo | CS | Adults on chronic liver disease or hepatoma | 354 | 47.2% | N/A |
| [Abdel-Wahab,94](file:///D:\Laith\Files\Pipeline\HCV\Yousra\Paper%20tables.xlsx#RANGE!_ENREF_60)[[52](#_ENREF_52)] | 1992 | Cairo city, Cairo | CS | Children with hepatosplenomegaly | 55 | 16.4% | N/A |
| El-Ahmady,94[[22](#_ENREF_22)] | N/A | N/A | CS |  | 102 | 50.0% | N/A |
| [Waked,95](file:///D:\Laith\Files\Pipeline\HCV\Yousra\Paper%20tables.xlsx#RANGE!_ENREF_55)[[53](#_ENREF_53)] | 1992 | Menoufia, Lower Egypt | CS |  | 1,023 | 73.5% | N/A |
| [Waked,95](file:///D:\Laith\Files\Pipeline\HCV\Yousra\Paper%20tables.xlsx#RANGE!_ENREF_55)[[53](#_ENREF_53)] | 1992 | Menoufia, Lower Egypt | CS | CLD patients: males | 645 | 79.1% | N/A |
| [Waked,95](file:///D:\Laith\Files\Pipeline\HCV\Yousra\Paper%20tables.xlsx#RANGE!_ENREF_55)[[53](#_ENREF_53)] | 1992 | Menoufia, Lower Egypt | CS | CLD patients: females | 378 | 64.0% | N/A |
| [Angelico,97](file:///D:\Laith\Files\Pipeline\HCV\Yousra\Paper%20tables.xlsx#RANGE!_ENREF_87)[[54](#_ENREF_54)] | 1993-95 | Rural villages, Alexandria | CS |  | 135 | 67.4% | 37.0% |
| [Madwar,97](file:///D:\Laith\Files\Pipeline\HCV\Yousra\Paper%20tables.xlsx#RANGE!_ENREF_105)[[55](#_ENREF_55)] | N/A | N/A | CS |  | 120 | 43.2% | N/A |
| [El-Medany,99](file:///D:\Laith\Files\Pipeline\HCV\Yousra\Paper%20tables.xlsx#RANGE!_ENREF_41)[[8](#_ENREF_8)] | N/A | Mansoura, Dakahlia, Lower Egypt | CS |  | 45 | 82.2% | N/A |
| [Khalifa,99](file:///D:\Laith\Files\Pipeline\HCV\Yousra\Paper%20tables.xlsx#RANGE!_ENREF_12)[[41](#_ENREF_41)] | N/A | N/A | CS |  | 61 | 56.0% | N/A |
| [Halim,99](file:///D:\Laith\Files\Pipeline\HCV\Yousra\Paper%20tables.xlsx#RANGE!_ENREF_42)[[6](#_ENREF_6)] | 1996 | Cairo city, Cairo | CS |  | 50 | 74.0% | N/A |
| [Gad,01](file:///D:\Laith\Files\Pipeline\HCV\Yousra\Paper%20tables.xlsx#RANGE!_ENREF_103)[[56](#_ENREF_56)] | 1998 | Ismailia, Lower Egypt | CS |  | 240 | 76.0% | N/A |
| [Strickland,02](file:///D:\Laith\Files\Pipeline\HCV\Yousra\Paper%20tables.xlsx#RANGE!_ENREF_84)[[57](#_ENREF_57)] | N/A | Nile River Delta, Lower Egypt | CS |  | 237 | 58.2% | 42.6% |
| [El-Zayadi,05](file:///D:\Laith\Files\Pipeline\HCV\Yousra\Paper%20tables.xlsx#RANGE!_ENREF_89)[[58](#_ENREF_58)] | 1993-2002 | Cairo city, Cairo | CS |  | 22,450 | 72.3% | N/A |
| [El Bassuoni,08](file:///D:\Laith\Files\Pipeline\HCV\Yousra\Paper%20tables.xlsx#RANGE!_ENREF_114)[[47](#_ENREF_47)] | N/A | Cairo city, Cairo | CS |  | 20 | 75.0% | N/A |
| [Zaki,11](file:///D:\Laith\Files\Pipeline\HCV\Yousra\Paper%20tables.xlsx#RANGE!_ENREF_73)[[59](#_ENREF_59)] | 2009-10 | Mansoura, Dakahlia, Lower Egypt | CS | Chronic liver failure patients | 100 | 100.0% | 30% |
|  |  |  |  |  |  |  |  |
| **Bladder cancer patients** | | | | | | | |
| [Yates,99](file:///D:\Laith\Files\Pipeline\HCV\Yousra\Paper%20tables.xlsx#RANGE!_ENREF_43)[[23](#_ENREF_23)] | N/A | Cairo city, Cairo | CS |  | 247 | 47.0% | N/A |
|  |  |  |  |  |  |  |  |
| **Rheumatic heart disease patients** | | | | | | | |
| [El-Nanawy,95](file:///D:\Laith\Files\Pipeline\HCV\Yousra\Paper%20tables.xlsx#RANGE!_ENREF_54)[[1](#_ENREF_1)] | N/A | Alexandria city, Alexandria | CS |  | 20 | 0.0% | N/A |
|  |  |  |  |  |  |  |  |
| **Chronic Renal Failure patients** | | | | | | | |
| [Gohar,95](file:///D:\Laith\Files\Pipeline\HCV\Yousra\Paper%20tables.xlsx#RANGE!_ENREF_58)[[33](#_ENREF_33)] | N/A | N/A | CS | Chronic renal insufficiency patients on conservative treatments | 15 | 53.3% | N/A |
| [El Yazeed,06](file:///D:\Laith\Files\Pipeline\HCV\Yousra\Paper%20tables.xlsx#RANGE!_ENREF_115)[[60](#_ENREF_60)] | 2002-04 | Cairo city, Cairo | CS | Renal impairment patients | 40 | 15.0% | N/A |
| [Hammad,09](file:///D:\Laith\Files\Pipeline\HCV\Yousra\Paper%20tables.xlsx#RANGE!_ENREF_72)[[61](#_ENREF_61)] | 2008 | Mansoura, Dakahlia, Lower Egypt | CS | Children with CRF | 100 | 52.0% | N/A |
| [Hammad,09](file:///D:\Laith\Files\Pipeline\HCV\Yousra\Paper%20tables.xlsx#RANGE!_ENREF_72)[[61](#_ENREF_61)] | 2008 | Mansoura, Dakahlia, Lower Egypt | CS | Children with CRF pre-dialysis | 66 | 30.3% | N/A |
|  |  |  |  |  |  |  |  |
| **Cancer patients** | | | | | | | |
| El-Ahmady,94[[22](#_ENREF_22)] | N/A | N/A | CS |  | 50 | 62.0% | N/A |
| [Attia,96](file:///D:\Laith\Files\Pipeline\HCV\Yousra\Paper%20tables.xlsx#RANGE!_ENREF_86)[[62](#_ENREF_62)] | N/A | Cairo city, Cairo | CS |  | 429 | 53.4% | N/A |
| [Mostafa,03](file:///D:\Laith\Files\Pipeline\HCV\Yousra\Paper%20tables.xlsx#RANGE!_ENREF_90)[[63](#_ENREF_63)] | 2000-07 | Cairo city, Cairo | CS | Newly diagnosed patients with pediatric malignancies (prior to starting treatment) | 111 | 0.9% | 0% |
| [Mostafa,03](file:///D:\Laith\Files\Pipeline\HCV\Yousra\Paper%20tables.xlsx#RANGE!_ENREF_90)[[63](#_ENREF_63)] | 2000-07 | Cairo city, Cairo | CS | Newly diagnosed patients with pediatric malignancies (after 6 months of chemotherapy) | 99 | 13.1% | 5.1% |
| [Mostafa,03](file:///D:\Laith\Files\Pipeline\HCV\Yousra\Paper%20tables.xlsx#RANGE!_ENREF_90)[[63](#_ENREF_63)] | 2000-07 | Cairo city, Cairo | CS | Patients with pediatric malignancies who ended chemotherapy | 111 | 39.6% | 18.9% |
| [Sharaf-Eldeen,07](file:///D:\Laith\Files\Pipeline\HCV\Yousra\Paper%20tables.xlsx#RANGE!_ENREF_126)[[64](#_ENREF_64)] | N/A | Cairo city, Cairo | CS | Children with malignant cancer | 100 | 43.0% | N/A |
|  |  |  |  |  |  |  |  |
| **Jaundice patients** | | | | | | | |
| [Hassan,93](file:///D:\Laith\Files\Pipeline\HCV\Yousra\Paper%20tables.xlsx#RANGE!_ENREF_63)[[17](#_ENREF_17)] | N/A | N/A | CS |  | 207 | 29.0% | N/A |
| [Gomatos,96](file:///D:\Laith\Files\Pipeline\HCV\Yousra\Paper%20tables.xlsx#RANGE!_ENREF_9)[[65](#_ENREF_65)] | 1993 | Cairo city, Cairo | CS |  | 219 | 8.4% | N/A |
| [Quinti,97](file:///D:\Laith\Files\Pipeline\HCV\Yousra\Paper%20tables.xlsx#RANGE!_ENREF_14)[[66](#_ENREF_66)] | N/A | Cairo city, Cairo | CS |  | 110 | 27.3% | 18.2% |
|  |  |  |  |  |  |  |  |
| **Patients suspected of having liver disease** | | | | | | | |
| [Takagi,03](file:///D:\Laith\Files\Pipeline\HCV\Yousra\Paper%20tables.xlsx#RANGE!_ENREF_127)[[67](#_ENREF_67)] | N/A | Alexandria city, Alexandria | CS |  | 57 | 64.9% | N/A |
| [Takagi,03](file:///D:\Laith\Files\Pipeline\HCV\Yousra\Paper%20tables.xlsx#RANGE!_ENREF_127)[[67](#_ENREF_67)] | N/A | Alexandria city, Alexandria | CS | Male | 45 | 82.2% | N/A |
| [Takagi,03](file:///D:\Laith\Files\Pipeline\HCV\Yousra\Paper%20tables.xlsx#RANGE!_ENREF_127)[[67](#_ENREF_67)] | N/A | Alexandria city, Alexandria | CS | Female | 12 | 83.3% | N/A |
| [Youssef,09](file:///D:\Laith\Files\Pipeline\HCV\Yousra\Paper%20tables.xlsx#RANGE!_ENREF_74)[[68](#_ENREF_68)] | N/A | Ismailia, Lower Egypt | CS | Individuals with elevated liver enzymes | 214 | 72.9% | 42.0% |
|  |  |  |  |  |  |  |  |
| **Patients with gastro intestinal bleeding** | | | | | | | |
| [Mikhail, 07](file:///D:\Laith\Files\Pipeline\HCV\Yousra\Paper%20tables.xlsx#RANGE!_ENREF_81)[[69](#_ENREF_69)] | 2000-04 | Cairo city, Cairo | CS | Patients undergoing diagnostic upper-GI endocscopy | 859 | 71.0% | N/A |
|  |  |  |  |  |  |  |  |
| **Meningitis patients** | | | | | | | |
| [Attallah,04](file:///D:\Laith\Files\Pipeline\HCV\Yousra\Paper%20tables.xlsx#RANGE!_ENREF_101)[[70](#_ENREF_70)] | N/A | Cairo city, Cairo | CS |  | 91 | 90.0% | N/A |
|  |  |  |  |  |  |  |  |
| **Patients with Organomegally** | | | | | | | |
| [Zaki,03](file:///D:\Laith\Files\Pipeline\HCV\Yousra\Paper%20tables.xlsx#RANGE!_ENREF_102)[[71](#_ENREF_71)] | 1998 | Abis village, Alexandria | CS | Individuals with clinically detected organomegally (swollen liver/spleen) | 65 | 33.8% | N/A |
|  |  |  |  |  |  |  |  |
| **Urological patients** | | | | | | | |
| [Demian,04](file:///D:\Laith\Files\Pipeline\HCV\Yousra\Paper%20tables.xlsx#RANGE!_ENREF_113)[[72](#_ENREF_72)] | N/A | Mansoura, Dakahlia, Lower Egypt | CS |  | 667 | 45.1% | N/A |
|  |  |  |  |  |  |  |  |
| **Systemic Lupus Erthematosus patients** | | | | | | | |
| [Kandil,07](file:///D:\Laith\Files\Pipeline\HCV\Yousra\Paper%20tables.xlsx#RANGE!_ENREF_119)[[73](#_ENREF_73)] | 2004-06 | Cairo city, Cairo | CS | Children with SLE | 15 | 40.0% | N/A |
|  |  |  |  |  |  |  |  |
| **Myelodysplastic Syndrome patients** | | | | | | | |
| [Mattar,11](file:///D:\Laith\Files\Pipeline\HCV\Yousra\Paper%20tables.xlsx#RANGE!_ENREF_121)[[74](#_ENREF_74)] | 2007-10 | Cairo city, Cairo | CS |  | 69 | 13.0% | N/A |
|  |  |  |  |  |  |  |  |
| **Glomerulonephritis patients** | | | | | | | |
| Abou-Zeid,11[[75](#_ENREF_75)] | N/A | Alexandria city, Alexandria | CS |  | 78 | 59.0% | N/A |
| CS = convenience sampling; N/A = not available; HCC = hepatocellular carcinoma; CLD = chronic liver disease; CRF = chronic renal failure; GI = gastrointestinal bleeding; SLE = systemic lupus erthematosus | | | | | | | |

**Table S4**- Hepatitis C virus time trend analysis for each general population subgroup

| Subgroup | Mean change in HCV prevalence  (95% Confidence Interval) | p-value |
| --- | --- | --- |
| Outpatient clinic attendees | -0.17 (-1.50, 1.17) | 0.720 |
| Antenatal clinic attendees | 0.00 (-0.83, 0.83) | 0.999 |
| Blood donors | -0.61 (-0.96, -0.26) | 0.001 |
| Rural village residents | 0.89 (-0.44, 2.21) | 0.178 |
| Children | -0.46 (-1.93, 1.02) | 0.315 |
| Healthy populations | -0.45 (-3.18, 2.29) | 0.711 |
| Army recruits/ Fire brigade personnel | 13.9 (-52.12, 79.92) | 0.228 |
| Other general populations | -0.32 (-1.85, 1.21) | 0.552 |

We conducted univariate linear regression analyses examining the trend in hepatitis C virus (HCV) prevalence over time in each subgroup of the general population separately. The results of the analyses are shown above. There is a slight decline in prevalence in several subgroups, however, this was found to be statistically significant only among blood donors. This decline nevertheless is difficult to interpret since recruitment of blood donors changed over time. Thus, the observed downward trend may not reflect a true reduction in prevalence, as much as a change in the selection criteria of blood donors, particularly by excluding HCV positive individuals.

**Figure S5-** Hepatitis C virus time trend analysis among populations at direct or high risk of exposure

**References**

1. el-Nanawy AA, el Azzouni OF, Soliman AT, Amer AE, Demian RS, el-Sayed HM: **Prevalence of hepatitis-C antibody seropositivity in healthy Egyptian children and four high risk groups**. *Journal of tropical pediatrics* 1995, **41**(6):341-343.

2. Zekri ARN, Sedkey L, El-Din HMA, Abdel-Aziz AO, Viazov S: **The pattern of transmission transfusion virus infection in Egyptian patients [6]**. *International Journal of Infectious Diseases* 2002, **6**(4):329-331.

3. Kandil ME, Rasheed MA, Saad NE: **Hepatitis C and B viruses among some high risk groups of Egyptian children**. *Journal of Medical Sciences* 2007, **7**(8):1259-1267.

4. Elmagd MM, Bakr MA, Metwally AH, Wahab AM: **Clinicoepidemiologic study of posttransplant diabetes after living-donor renal transplant**. *Experimental and clinical transplantation : official journal of the Middle East Society for Organ Transplantation* 2008, **6**(1):42-47.

5. El-Karaksy H, Anwar GH, El-Raziky MS, El-Hawary M, Hashem M, El-Sayed R, El-Shabrawi M, Mohsen N, Fouad H, Esmat G: **Anti-HCV prevalence among diabetic and non-diabetic Egyptian children**. *Current diabetes reviews* 2010, **6**(6):388-392.

6. Halim AB, Garry RF, Dash S, Gerber MA: **Effect of schistosomiasis and hepatitis on liver disease**. *The American journal of tropical medicine and hygiene* 1999, **60**(6):915-920.

7. Kalil KA, Farghally HS, Hassanein KM, Abd-Elsayed AA, Hassanein FE: **Hepatitis C virus infection among paediatric patients attending University of Assiut Hospital, Egypt**. *Eastern Mediterranean health journal = La revue de sante de la Mediterranee orientale = al-Majallah al-sihhiyah li-sharq al-mutawassit* 2010, **16**(4):356-361.

8. El-Medany OM, El-Din Abdel Wahab KS, Abu Shady EA, Gad El-Hak N: **Chronic liver disease and hepatitis C virus in Egyptian patients**. *Hepato-gastroenterology* 1999, **46**(27):1895-1903.

9. Agha S, Sherif LS, Allam MA, Fawzy M: **Transplacental transmission of hepatitis C virus in HIV-negative mothers**. *Research in virology* 1998, **149**(4):229-234.

10. Kassem AS, El-Nawawy AA, Massoud MN, Abou El-Nazar SY, Sobhi EM: **Prevalence of hepatitis C virus (HCV) infection and its vertical transmission in Egyptian pregnant women and their newborns**. *Journal of tropical pediatrics* 2000, **46**(4):231-233.

11. Shebl FM, El-Kamary SS, Saleh DA, Abdel-Hamid M, Mikhail N, Allam A, El-Arabi H, Elhenawy I, El-Kafrawy S, El-Daly M *et al*: **Prospective cohort study of mother-to-infant infection and clearance of hepatitis C in rural Egyptian villages**. *Journal of medical virology* 2009, **81**(6):1024-1031.

12. Zahran KM, Badary MS, Agban MN, Abdel Aziz NH: **Pattern of hepatitis virus infection among pregnant women and their newborns at the Women's Health Center of Assiut University, Upper Egypt**. *International journal of gynaecology and obstetrics: the official organ of the International Federation of Gynaecology and Obstetrics* 2010, **111**(2):171-174.

13. AbdulQawi K, Youssef A, Metwally MA, Ragih I, AbdulHamid M, Shaheen A: **Prospective study of prevalence and risk factors for hepatitis C in pregnant Egyptian women and its transmission to their infants**. *Croatian medical journal* 2010, **51**(3):219-228.

14. Abo Elmagd EK, Abdel-Wahab KS, Alrasheedy ZE, Khalifa AS: **An Egyptian study of mother to child transmission of hepatitis C virus**. *International Journal of Virology* 2011, **7**(3):100-108.

15. El-Zayadi A, Khalifa AA, El-Misiery A, Naser AM, Dabbous H, Aboul-Ezz AA: **Evaluation of risk factors for intrafamilial transmission of HCV infection in Egypt**. *The Journal of the Egyptian Public Health Association* 1997, **72**(1-2):33-51.

16. Morad WS: **Transmission of hepatitis C between spouses an epidemiological study at National Liver Institute hospital**. *International Journal of Infectious Diseases* 2011, **15**:S81.

17. Hassan NF: **Prevalence of hepatitis C antibodies in patient groups in Egypt**. *Transactions of the Royal Society of Tropical Medicine and Hygiene* 1993, **87**(6):638.

18. Ali F, Abdel-Aziz A, Helmy MF, Abdel-Mobdy A, Darwish M: **Prevalence of certain sexually transmitted viruses in Egypt**. *The Journal of the Egyptian Public Health Association* 1998, **73**(3-4):181-192.

19. Quinti I, Renganathan E, El Ghazzawi E, Divizia M, Sawaf G, Awad S, Pana A, Rocchi G: **Seroprevalence of HIV and HCV infections in Alexandria, Egypt**. *Zentralblatt fur Bakteriologie : international journal of medical microbiology* 1995, **283**(2):239-244.

20. Shalaby S, Kabbash IA, El Saleet G, Mansour N, Omar A, El Nawawy A: **Hepatitis B and C viral infection: prevalence, knowledge, attitude and practice among barbers and clients in Gharbia governorate, Egypt**. *Eastern Mediterranean health journal = La revue de sante de la Mediterranee orientale = al-Majallah al-sihhiyah li-sharq al-mutawassit* 2010, **16**(1):10-17.

21. Hindy AM, Abdelhaleem ES, Aly RH: **Hepatitis B and C viruses among Egyptian dentists**. *Egyptian dental journal* 1995, **41**(3):1217-1226.

22. el-Ahmady O, Halim AB, Mansour O, Salman T: **Incidence of hepatitis C virus in Egyptians**. *Journal of hepatology* 1994, **21**(4):687.

23. Yates SC, Hafez M, Beld M, Lukashov VV, Hassan Z, Carboni G, Khaled H, McMorrow M, Attia M, Goudsmit J: **Hepatocellular carcinoma in Egyptians with and without a history of hepatitis B virus infection: Association with hepatitis C virus (HCV) infection but not with HCV RNA level**. *American Journal of Tropical Medicine and Hygiene* 1999, **60**(4):714-720.

24. Abdelwahab S, Rewisha E, Hashem M, Sobhy M, Galal I, Allam WR, Mikhail N, Galal G, El-Tabbakh M, El-Kamary SS *et al*: **Risk factors for hepatitis C virus infection among Egyptian healthcare workers in a national liver diseases referral centre**. *Transactions of the Royal Society of Tropical Medicine and Hygiene* 2012, **106**(2):98-103.

25. Farghaly AG, Mansour GA, Mahdy NH, Yousri A: **Hepatitis B and C virus infections among patients with gingivitis and adult periodontitis: seroprevalence and public health importance**. *The Journal of the Egyptian Public Health Association* 1998, **73**(5-6):707-735.

26. Cowgill KD, Loffredo CA, Eissa SA, Mokhtar N, Abdel-Hamid M, Fahmy A, Strickland GT: **Case-control study of non-Hodgkin's lymphoma and hepatitis C virus infection in Egypt**. *International journal of epidemiology* 2004, **33**(5):1034-1039.

27. El-Sayed GM, Mohamed WS, Nouh MA, Moneer MM, El-Mahallawy HA: **Viral genomes and antigen detection of hepatitis B and C viruses in involved lymph nodes of Egyptian non-Hodgkin's lymphoma patients**. *The Egyptian journal of immunology / Egyptian Association of Immunologists* 2006, **13**(1):105-114.

28. Goldman L, Ezzat S, Mokhtar N, Abdel-Hamid A, Fowler N, Gouda I, Eissa SA, Abdel-Hamid M, Loffredo CA: **Viral and non-viral risk factors for non-Hodgkin's lymphoma in Egypt: heterogeneity by histological and immunological subtypes**. *Cancer Causes Control* 2009, **20**(6):981-987.

29. Kassem N, Hamada E, Lasheen SH, Metwally R: **Hepatitis c infection and non hodgkin lymphoma in 37 Egyptian patients: A single institute experience**. *Haematologica* 2009, **Conference: 14th Congress of the European Hematology Association Berlin Germany. Conference Start: 20090604 Conference End: 20090607. Conference Publication: (var.pagings). 94**:656.

30. Farawela H, Khorshied M, Shaheen I, Gouda H, Nasef A, Abulata N, Mahmoud HA, Zawam HM, Mousa SM: **The association between hepatitis C virus infection, genetic polymorphisms of oxidative stress genes and B-cell non-Hodgkin's lymphoma risk in Egypt**. *Infection, Genetics and Evolution* 2012, **12**(6):1189-1194.

31. Ezzat S, Abdel-Hamid M, Eissa SA, Mokhtar N, Labib NA, El-Ghorory L, Mikhail NN, Abdel-Hamid A, Hifnawy T, Strickland GT *et al*: **Associations of pesticides, HCV, HBV, and hepatocellular carcinoma in Egypt**. *International journal of hygiene and environmental health* 2005, **208**(5):329-339.

32. Abdel Wahab M, Mostafa M, Salah T, Fouud A, Kandeel T, Elshobary M, Abd Allah OF, Elghawalby N, Sultan A, Ezzat F: **Epidemiology of hilar cholangiocarcinoma in Egypt: single center study**. *Hepato-gastroenterology* 2007, **54**(78):1626-1631.

33. Gohar SA, Khalil RY, Elaish NM, Khedr EM, Ahmed MS: **Prevalence of antibodies to hepatitis C virus in hemodialysis patients and renal transplant recipients**. *The Journal of the Egyptian Public Health Association* 1995, **70**(5-6):465-484.

34. Sabry A, Hassan R, Mahmoud I, Hamed M, Sobh M: **Proteinuria after kidney transplantation: its relation to hepatitis C virus and graft outcome**. *Iranian journal of kidney diseases* 2007, **1**(2):88-97.

35. Ibrahim HA, Baddour MM, Morsi MG, Abdelkader AA: **Should we routinely check for hepatitis B and C in patients with lichen planus or cutaneous vasculitis?** *Eastern Mediterranean health journal = La revue de sante de la Mediterranee orientale = al-Majallah al-ihhiyah li-sharq al-mutawassi* 1999, **5**(1):71-78.

36. Amer MA, El-Harras M, Attwa E, Raslan S: **Lichen planus and hepatitis C virus prevalence and clinical presentation in Egypt**. *Journal of the European Academy of Dermatology and Venereology : JEADV* 2007, **21**(9):1259-1260.

37. Zekri ARN, Sedkey L, El-Din HMA, Abdel-Aziz AO, Viazov S: **The pattern of transmission transfusion virus infection in Egyptian patients [6]**. *International Journal of Infectious Diseases* 2002, **6 (4)**:329-331.

38. Darwish MA, Issa SA, Aziz AM, Darwish NM, Soliman AH: **Hepatitis C and B viruses, and their association with hepatocellular carcinoma in Egypt**. *The Journal of the Egyptian Public Health Association* 1993, **68**(1-2):1-9.

39. Mabrouk GM: **Prevalence of hepatitis C infection and schistosomiasis in Egyptian patients with hepatocellular carcinoma**. *Disease markers* 1997, **13**(3):177-182.

40. Darwish MA, Amer AF, El-Moeity AA, Darwish NM: **Association of hepatitis C virus with liver cirrhosis and hepatocellular carcinoma compared with hepatitis B virus in Egyptian patients**. *The Journal of the Egyptian Public Health Association* 1997, **72**(5-6):569-589.

41. Khalifa A, Mady EA, Abadeer N, Kamal A: **Differential tumor markers and hepatitis markers profile in liver tumors**. *Anticancer Research* 1999, **19**(4 A):2495-2500.

42. Abdel-Wahab M, El-Enein AA, Abou-Zeid M, El-Fiky A, Abdallah T, Fawzy M, Fouad A, Sultan A, Fathy O, El-Ebidy G *et al*: **Hepatocellular carcinoma in Mansoura - Egypt: Experience of 385 patients at a single center**. *Hepato-gastroenterology* 2000, **47**(33):663-668.

43. Hassan MM, Zaghloul AS, El-Serag HB, Soliman O, Patt YZ, Chappell CL, Beasley RP, Hwang LY: **The role of hepatitis C in hepatocellular carcinoma: A case control study among egyptian patients**. *Journal of Clinical Gastroenterology* 2001, **33**(2):123-126.

44. Rahman El-Zayadi A, Abaza H, Shawky S, Mohamed MK, Selim OE, Badran HM: **Prevalence and epidemiological features of hepatocellular carcinoma in Egypt - A single center experience**. *Hepatology Research* 2001, **19 (2)**:170-179.

45. Abdel-Wahab M, El-Ghawalby N, Mostafa M, Sultan A, El-Sadany M, Fathy O, Salah T, Ezzat F: **Epidemiology of hepatocellular carcinoma in lower Egypt, Mansoura Gastroenterology Center**. *Hepato-gastroenterology* 2007, **54**(73):157-162.

46. Abdel-Wahab M, Mostafa M, Sabry M, El-Farrash M, Yousef T: **Aflatoxins as a risk factor for hepatocellular carcinoma in Egypt, Mansoura Gastroenterology Center Study**. *Hepato-gastroenterology* 2008, **55**(86-87):1754-1759.

47. El Bassuoni MA, Obada MA, Korah T, El Sayed S: **Assessment of Treg cells CD4+CD25+ in chronic cirrhotic liver disease and hepatocellular carcinoma Egyptian patients**. *Hepatitis Monthly* 2008, **8 (3)**:173-177.

48. Abdel-Maksoud S, Mansour MT, Ali OSM, El Bassyouni HT, El Sayed El Awady RR: **Hepatitis C virus infection and gene expression in hepatocellular carcinoma patients**. *Basic and Clinical Pharmacology and Toxicology* 2009, **Conference: 9th Congress of the European Association for Clinical Pharmacology and Therapeutics Edinburgh United Kingdom. Conference Start: 20090712 Conference End: 20090715. Conference Publication: (var.pagings). 105**:111.

49. Taha A, Hasan M, El-Ray A, El-Ghannam M, Helmy AH, Esmat E, Yilmaz N: **Clinicoepidemiological characteristics and response to treatment in patients with hepatocellular carcinoma in Egypt**. *Hepatology International* 2012, **6 (1)**:218.

50. Meir H, Balawi I, Nayel H, El Karaksy H, El Haddad A: **Hepatic dysfunction in children with acute lymphoblastic leukemia in remission: relation to hepatitis infection**. *Medical and pediatric oncology* 2001, **36**(4):469-473.

51. el-Zayadi A, Selim O, Rafik M, el-Haddad S: **Prevalence of hepatitis C virus among non-A, non-B-related chronic liver disease in Egypt**. *Journal of hepatology* 1992, **14**(2-3):416-417.

52. Abdel-Wahab MF, Zakaria S, Kamel M, Abdel-Khaliq MK, Mabrouk MA, Salama H, Esmat G, Thomas DL, Strickland GT: **High seroprevalence of hepatitis C infection among risk groups in Egypt**. *The American journal of tropical medicine and hygiene* 1994, **51**(5):563-567.

53. Waked IA, Saleh SM, Moustafa MS, Raouf AA, Thomas DL, Strickland GT: **High prevalence of hepatitis C in Egyptian patients with chronic liver disease**. *Gut* 1995, **37**(1):105-107.

54. Angelico M, Renganathan E, Gandin C, Fathy M, Profili MC, Refai W, De Santis A, Nagi A, Amin G, Capocaccia L *et al*: **Chronic liver disease in the Alexandria governorate, Egypt: contribution of schistosomiasis and hepatitis virus infections**. *Journal of hepatology* 1997, **26**(2):236-243.

55. Madwar MA, Shaker MK, Atta MA, El Khashaab TH, Mohamed MK: **A prospective study: prediction of the first variceal haemorrhage in schistosomal and non schistosomal liver disease**. *The Journal of the Egyptian Public Health Association* 1997, **72**(3-4):395-409.

56. Gad A, Tanaka E, Orii K, Rokuhara A, Nooman Z, Serwah AH, Shoair M, Yoshizawa K, Kiyosawa K: **Relationship between hepatitis C virus infection and schistosomal liver disease: not simply an additive effect**. *Journal of gastroenterology* 2001, **36**(11):753-758.

57. Strickland GT, Elhefni H, Salman T, Waked I, Abdel-Hamid M, Mikhail NN, Esmat G, Fix A: **Role of hepatitis C infection in chronic liver disease in Egypt**. *The American journal of tropical medicine and hygiene* 2002, **67**(4):436-442.

58. el-Zayadi AR, Badran HM, Barakat EM, Attia Mel D, Shawky S, Mohamed MK, Selim O, Saeid A: **Hepatocellular carcinoma in Egypt: a single center study over a decade**. *World journal of gastroenterology : WJG* 2005, **11**(33):5193-5198.

59. El Sayed Zaki M, Othman W: **Role of hepatitis E infection in acute on chronic liver failure in Egyptian patients**. *Liver international : official journal of the International Association for the Study of the Liver* 2011, **31**(7):1001-1005.

60. El Yazeed SA, N.A EL-G, Younes K, El-Ghobary A: **Antiphospholipid antibodies in Egyptian patients with chronic renal failure**. *Journal of Medical Sciences* 2006, **6 (3)**:468-473.

61. Hammad AM, Zaghloul MH: **Hepatitis G virus infection in Egyptian children with chronic renal failure (single centre study)**. *Annals of clinical microbiology and antimicrobials* 2009, **8**:36.

62. Attia MA, Zekri AR, Goudsmit J, Boom R, Khaled HM, Mansour MT, de Wolf F, el-Din HM, Sol CJ: **Diverse patterns of recognition of hepatitis C virus core and nonstructural antigens by antibodies present in Egyptian cancer patients and blood donors**. *Journal of clinical microbiology* 1996, **34**(11):2665-2669.

63. Mostafa A, Ebeid, E., Mansour, T. , Amin, M., Sidhom, I. Khairy, A. El Zomor, H.: **Seroprevalence of Hepatitis B and C in Pediatric Malignancies**. *Journal of the Egyptian Nat Cancer Inst* 2003, **15**(1):33-42.

64. Sharaf-Eldeen S, Salama K, Eldemerdash S, Hassan HMS, Semesem M: **Hepatitis B and C Viruses in Egyptian children with malignancy**. *Journal of Medical Sciences* 2007, **7 (6)**:1003-1008.

65. Gomatos PJ, Monier MK, Arthur RR, Rodier GR, el-Zimaity D, Hassan NF, Quinti I, el-Sahly AD, Sultan Y, Hyams KC: **Sporadic acute hepatitis caused by hepatitis E virus in Egyptian adults**. *Clinical infectious diseases : an official publication of the Infectious Diseases Society of America* 1996, **23**(1):195-196.

66. Quinti I, El Salman D, Monier MK, Hackbart BG, Darwish MS, El-Zamiaty D, Paganelli R, Pandolfi F, Arthur RR: **HCV infection in Egyptian patients with acute hepatitis**. *Digestive Diseases and Sciences* 1997, **42**(10):2017-2023.

67. Takagi H, Toyoda M, Sohara N, Otsuka T, Kakizaki S, Mori M: **Liver disease in Alexandria, Egypt**. *Kitakanto Medical Journal* 2003, **53 (2)**:175-177.

68. Youssef A, Yano Y, Utsumi T, abd El-alah EM, abd El-Hameed Ael E, Serwah Ael H, Hayashi Y: **Molecular epidemiological study of hepatitis viruses in Ismailia, Egypt**. *Intervirology* 2009, **52**(3):123-131.

69. Mikhail NN, Lewis DL, Omar N, Taha H, El-Badawy A, Abdel-Mawgoud N, Abdel-Hamid M, Strickland GT: **Prospective study of cross-infection from upper-GI endoscopy in a hepatitis C-prevalent population**. *Gastrointestinal endoscopy* 2007, **65**(4):584-588.

70. Attallah AM, Ibrahim GG: **Immunodetection of a hepatitis C virus (HCV) antigen and Thl/Th2 cytokines in cerebrospinal fluid of meningitis patients**. *Journal of immunoassay & immunochemistry* 2004, **25**(4):313-320.

71. Zaki A, Bassili A, Amin G, Aref T, Kandil M, Abou Basha LM: **Morbidity of schistosomiasis mansoni in rural Alexandria, Egypt**. *Journal of the Egyptian Society of Parasitology* 2003, **33**(3):695-710.

72. Demian AD: **Prevalence of anaesthetic co-morbid factors among urological patients in a tertiary referral centre in Egypt**. *Egyptian Journal of Anaesthesia* 2004, **20 (3)**:325-330.

73. Kandil ME, Rasheed MA, Saad NE: **Hepatitis C and B viruses among some high risk groups of Egyptian children**. *Journal of Medical Sciences* 2007, **7 (8)**:1259-1267.

74. Mattar M, El Husseiny N, Asaad S: **Myelodysplastic syndrome. Egyptian experience**. *Leukemia Research* 2011, **Conference: 11th International Symposium on Myelodysplastic Syndromes, MDS Edinburgh United Kingdom. Conference Start: 20110518 Conference End: 20110521. Conference Publication: (var.pagings). 35**:S146.

75. Abou-Zeid AA, El-Sayegh HK: **Toll-like receptor 3 gene expression in Egyptian patients with glomerulonephritis and hepatitis C virus infection**. *Scandinavian journal of clinical and laboratory investigation* 2011, **71**(6):456-461.
